# *CYBA* Gene Polymorphisms, Lipid Parameters and Atherogenic Indices: A Cohort Study of Healthy Individuals Without Prior Lipid-Lowering Therapy

**DOI:** 10.3390/ijms27208975

**Published:** 2026-10-10

**Authors:** Paweł Niemiec, Tomasz Nowak, Justyna Wrona, Joanna Iwanicka, Tomasz Iwanicki, Alicja Jarosz, Sylwia Górczyńska-Kosiorz, Anna Balcerzyk-Matić

**Affiliations:** 1 Department of Biochemistry and Medical Genetics, Faculty of Health Sciences in Katowice, Medical University of Silesia, Medykow Street 18, 40-752 Katowice, Poland; tnowak@sum.edu.pl (T.N.); justyna.wrona@sum.edu.pl (J.W.); jiwanicka@sum.edu.pl (J.I.); tiwanicki@sum.edu.pl (T.I.); alicja.jarosz@sum.edu.pl (A.J.); abalcerzyk@sum.edu.pl (A.B.-M.); 2 Department of Internal Medicine, Diabetology and Nephrology, Faculty of Medical Sciences in Zabrze, Medical University of Silesia, 40-055 Katowice, Poland; skosiorz@sum.edu.pl

**Keywords:** NADPH oxidases, p22phox, *CYBA* gene polymorphisms, lipid profile

## Abstract

Single-nucleotide polymorphisms (SNPs) in the *CYBA* gene, encoding the p22phox subunit of vascular NADPH oxidases, have been associated with atherosclerosis-related disorders. This association may partly reflect their involvement in lipid metabolism. We investigated the effects of seven *CYBA* SNPs (rs7195830, rs1049255, rs4673, rs13306296, rs72811418, rs16966671, and rs9932581) on systemic lipid profiles (total cholesterol, HDL-C, LDL-C, and triglycerides) and six lipid-derived atherogenic indices in 220 blood donors who had never received lipid-lowering therapy. Genotyping was performed using PCR-RFLP and TaqMan PCR. Serum lipid concentrations were determined by enzymatic colorimetric methods. For all SNPs, in silico analyses assessed allele-specific binding (ASB) of transcription factors (TFs) and gene expression in cardiovascular tissues. Statistically significant differences in lipid parameters were observed among the rs7195830, rs1049255, and rs13306296 variants. In silico analyses indicated ASB of TFs for rs1049255 and rs13306296 and genotype-dependent effects of rs13306296 and rs4673 on *CYBA* expression. Notably, the G allele/GG genotype of rs13306296 was associated with the least favorable lipid profile across multiple parameters, preferential binding of thirteen TFs, and increased *CYBA* expression in aorta and heart tissues. In conclusion, *CYBA* variants influence the systemic lipid profile and lipid-derived atherogenic markers in individuals untreated with lipid-lowering drugs, potentially through modulation of *CYBA* expression.

## 1. Introduction

NADPH oxidases are multicomponent enzymes responsible for the generation of superoxide anion and represent a major source of reactive oxygen species (ROS) in the cardiovascular system [1,2,3,4,5,6]. The catalytic subunit of these complexes consists of membrane-bound NOX (NOX1–NOX5) or DUOX (DUOX1 and DUOX2) proteins, which, together with a range of other proteins, including phox (phagocyte oxidase), Rac (Rho family small GTP-binding proteins), and other regulatory components, form functional enzyme complexes [1,2,3]. By generating ROS, whose biological effects depend on both their levels and the cellular context, NADPH oxidases play important roles in physiological and pathological processes. They are essential for host defense against microorganisms through the generation of the respiratory burst [2,3] and regulate signal transduction, gene expression, vascular tone, cell differentiation, and tissue adaptive responses [1,4,5,6]. However, chronic, excessive, or dysregulated activation of NADPH oxidases leads to oxidative stress, which plays an important role in the pathogenesis of atherosclerosis, contributing to endothelial dysfunction, hypertension, initiation of inflammatory responses, lipoprotein oxidation, and the development of acute cardiovascular events [1,2,3,4,5,6].

The membrane protein p22phox is a common component of NADPH oxidase complexes containing NOX1–NOX4 and is expressed in key cell types of the cardiovascular system, including vascular smooth muscle cells, cardiomyocytes, endothelial cells, and fibroblasts [1]. Increased vascular p22phox expression is associated with excessive ROS production and reduced nitric oxide bioavailability, thereby contributing to vascular endothelial dysfunction [7,8,9]. p22phox is also markedly overexpressed in atherosclerotic plaques, particularly in macrophage- and smooth muscle cell-rich areas, where it contributes to local inflammatory responses, plaque destabilization, and increased susceptibility to rupture [10,11,12]. Increased p22phox expression has also been demonstrated in acute cardiovascular events, including myocardial infarction [7,13,14], as well as in ischemic stroke [15,16]. The p22phox protein is encoded by the *CYBA* gene (16q24), which, unlike genes encoding other NADPH oxidase subunits, is characterized by substantial genetic polymorphism. Single-nucleotide polymorphisms (SNPs) in *CYBA* include, among others, rs4673 (214 C>T, C242T), rs1049255 (*24 A>G, A640G), and rs9932581 (−930 A>G). The first two SNPs have been the most extensively investigated in the context of atherosclerotic diseases and have been examined in meta-analyses documenting their associations with, among other conditions, hypertension [17], coronary artery disease (CAD) [18,19,20], and ischemic stroke [21,22].

Because atherosclerosis and its clinical consequences are multifactorial disorders [23,24], oxidative stress interacts with numerous other risk factors within the biological system to determine the disease phenotype. Among the major traditional risk factors for atherosclerosis and CAD are abnormalities in lipid metabolism. The oxidative modification of low-density lipoprotein cholesterol (LDL-C) to oxidized LDL (oxLDL), mediated by ROS generated by NADPH oxidases, is considered a well-established key step in atherogenesis [10,14]. Interestingly, evidence also supports a reverse relationship, namely, a positive feedback loop between oxLDL exposure and the expression of *NOX4* (mRNA and protein) and p22phox in macrophages [25]. These are not the only known interactions between ROS and lipid metabolism. For example, exposure of hepatocytes to H_2_O_2_ has been shown to increase the activity and expression of key enzymes involved in fatty acid and cholesterol synthesis, including acetyl-CoA carboxylase, fatty acid synthase, and HMG-CoA reductase, as well as the expression of the transcription factors SREBP-1 and SREBP-2, which are involved in the activation of lipid biosynthesis, accompanied by increased intracellular triacylglycerol (TG) content [26,27]. Similarly, human umbilical artery endothelial cells exposed to H_2_O_2_ exhibited increased cholesterol levels, NADPH oxidase activity, and *NOX4* expression (mRNA and protein) [28]. In another study, mice with myeloid cell-specific deficiency of NOX2 exhibited significantly lower serum concentrations of total cholesterol (TC), LDL-C, and triacylglycerols, together with significantly higher high-density lipoprotein cholesterol (HDL-C) levels, compared with control mice [29].

Despite accumulating evidence implicating NADPH oxidases in oxidative lipid modifications and lipid metabolism, the available literature contains only limited and fragmented evidence regarding the potential effects of genetic profiles of their constituent subunits on the systemic lipid profile. Therefore, in the present study, we analyzed serum lipid concentrations, including TC, LDL-C, HDL-C, and TG, in healthy blood donors with no history of lipid-lowering treatment, in relation to genotype variants of seven *CYBA* gene SNPs (rs7195830, rs1049255, rs4673, rs13306296, rs72811418, rs16966671, and rs9932581), including six located within the regulatory regions of the gene (promoter and 3′ UTR). The present study also aimed to investigate the associations between *CYBA* polymorphisms and six well-established lipid-derived atherogenic indices, as well as in silico analyses of allele-specific binding of transcription factors and *CYBA* gene expression in cardiovascular tissues.

## 2. Results

### 2.1. General Characteristics of the Study Group

The study cohort consisted of 220 blood donors, with a median age of 44 years (±4.50). Nearly 70% of the participants were male. Increased body mass index (BMI ≥ 25) was observed in 60% of the subjects, of whom approximately 15% were obese (BMI ≥ 30). Median concentrations of TC, HDL-C, LDL-C, and TG indicated a high prevalence of lipid metabolism disorders within the study group, which may partly be explained by the fact that these individuals had never received treatment for dyslipidemia prior to enrollment. A small proportion of the blood donors had hypertension (approximately 2%), and about 25% were active smokers of conventional cigarettes (the group was recruited before the rise in popularity of e-cigarettes and other substitutes, such as heated tobacco products). Table 1 presents the basic demographic and biochemical characteristics of the study participants, including lipid-derived atherogenic indices.

Sex, hypertension status, smoking status, and the presence of overweight or obesity did not significantly affect serum lipid parameters or atherogenic index values (*p* > 0.050). Likewise, no statistically significant correlations were found between serum lipid concentrations or atherogenic indices and either age or BMI of the participants.

### 2.2. CYBA Gene Polymorphisms

Data obtained from the genotyping of seven *CYBA* gene polymorphisms, along with the chromosomal locations of the respective SNPs, are presented in Table 2. The observed genotype distribution for all analyzed polymorphisms did not significantly deviate from the expected values and was consistent with Hardy–Weinberg equilibrium (*p* > 0.050).

No significant differences in age were observed among the genotypes of the analyzed SNPs, with the exception of the rs16966671 polymorphism (–852 C>G); however, this difference (*p* = 0.047) was observed only before the correction for multiple comparisons. The distribution of sex, as well as the prevalence of overweight/obesity and smoking status, was comparable across genotypes (*p* > 0.050).

**Figure 1:**
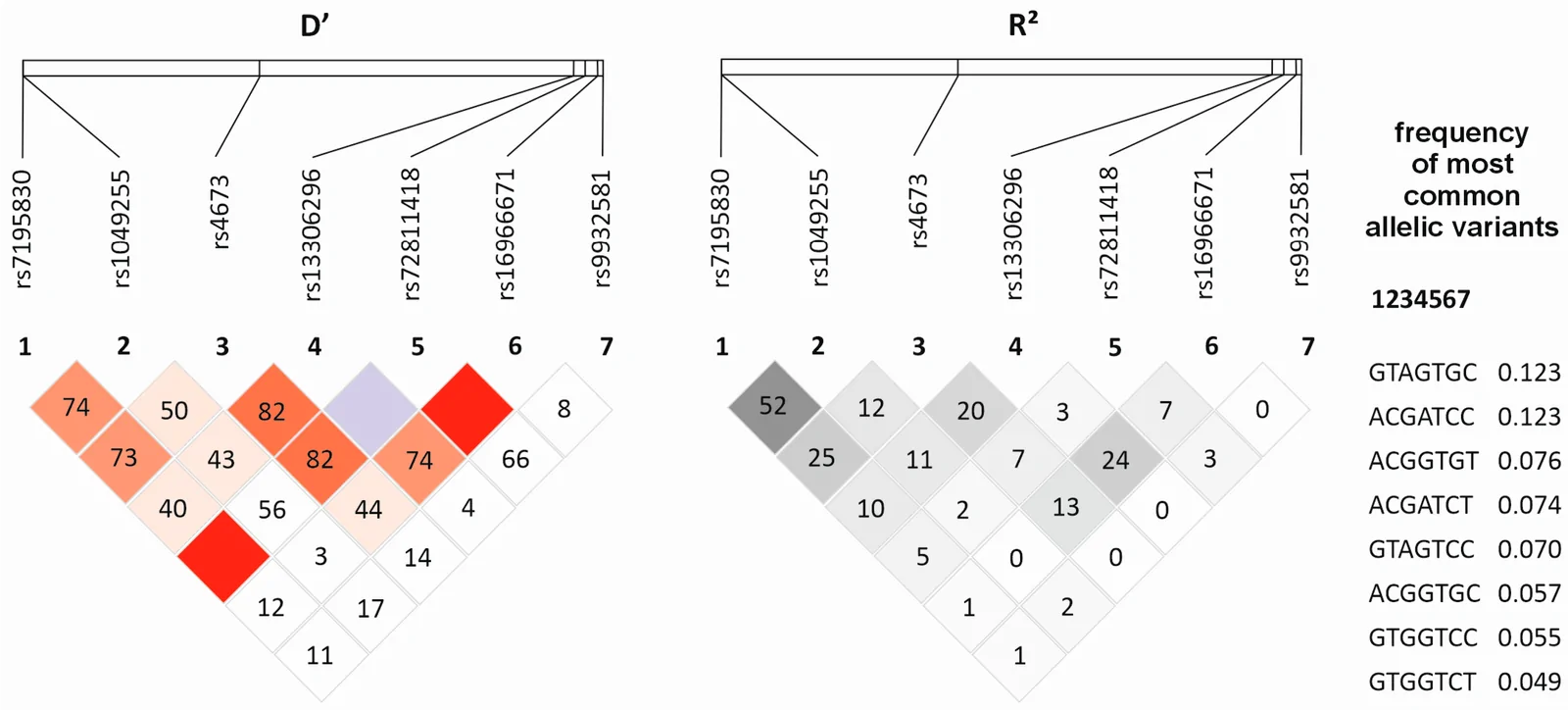
Haplotype analysis of the *CYBA* gene polymorphisms in the study group.

No haplotype blocks were identified among the seven analyzed *CYBA* variants according to the algorithm used (see Section 4), which is confirmed by the data for Caucasian populations [31]. Although several haplotypes were reconstructed, pairwise linkage disequilibrium was generally limited, particularly in terms of R^2^ (Figure 1). The most frequent reconstructed haplotypes were GTAGTGC and ACGATCC, each with an estimated frequency of 0.123; however, no predominant haplotype structure encompassing the analyzed variants was observed.

### 2.3. CYBA Gene Polymorphisms, Serum Lipid Parameters and Lipid-Derived Atherogenic Indices

In both the additive and dominant/recessive models, numerous associations were demonstrated between genotypic variants of the *CYBA* gene and serum lipid parameters as well as atherogenic indices in a univariate model. After adjustment for multiple comparisons, the statistical significance of differences in the distribution of analyzed parameters remained only for the polymorphic variants rs7195830 (*49 A>G), rs1049255 (*24 A>G, A640G), and rs13306296 (–536 C>T).

**Figure 2:**
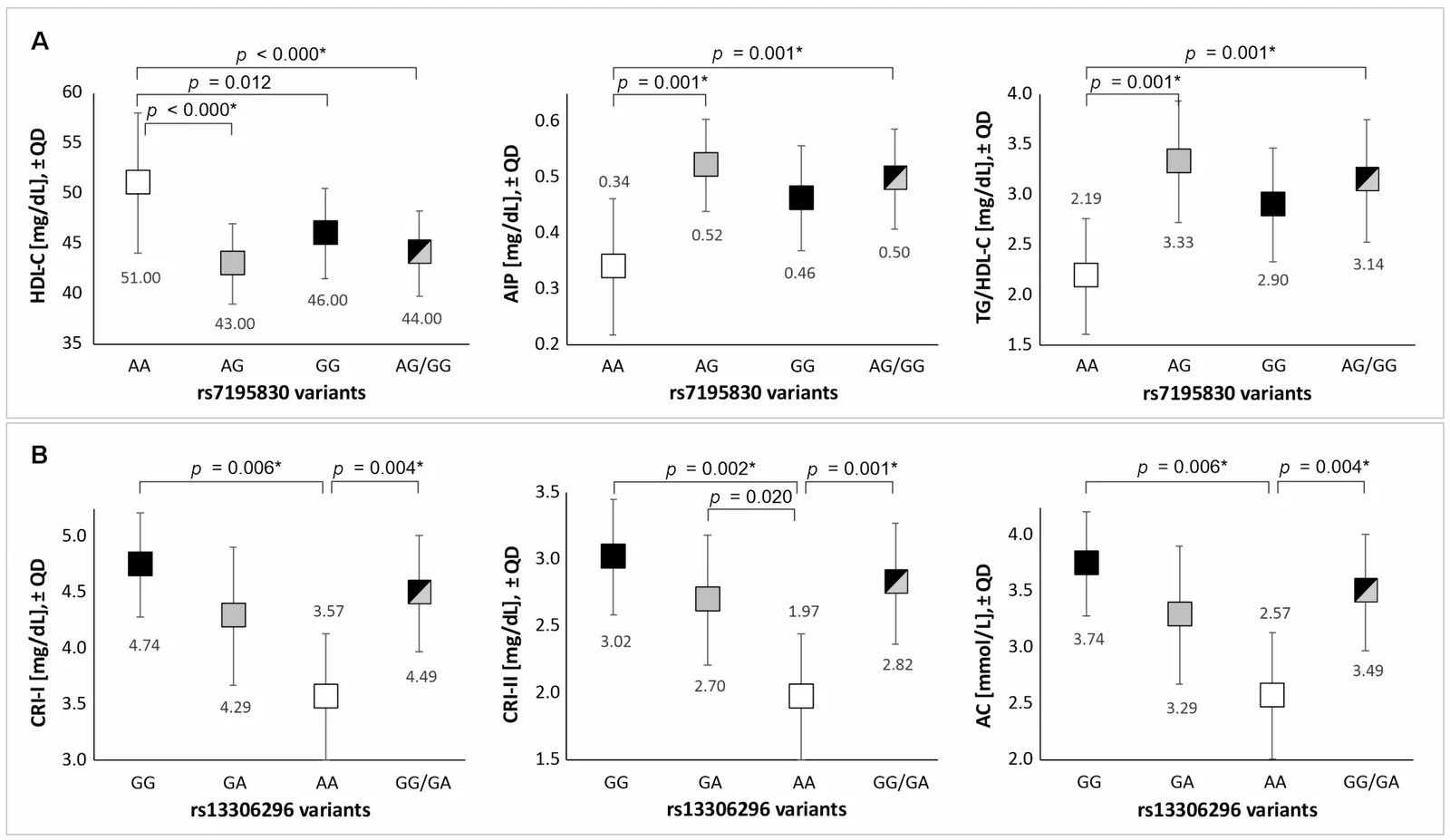
Comparison of lipid levels (HDL-C) and values of proatherogenic indices among *CYBA* gene variants in the dominant/recessive model (univariate analysis): (**A**) for rs7195830; and (**B**) for the rs13306296 SNP. *, differences remaining significant after Bonferroni–Hochberg correction for multiple comparisons (threshold of significance: for the additive model: *p* ≤ 0.008; for the dominant/recessive model: *p* ≤ 0.004).

Homozygotes with the AA genotype of the rs7195830 polymorphism exhibited significantly higher HDL-C concentrations compared with both AG heterozygotes, G allele carriers and GG homozygotes, although the latter difference was significant only before correction for multiple comparisons (Figure 2A). Additionally, AA homozygotes (rs7195830) showed lower values of the atherogenic indices AIP and TG/HDL-C compared with AG heterozygotes and G allele carriers (Figure 2A). In contrast, TT homozygotes of the rs1049255 polymorphism had significantly lower HDL-C concentrations than CC homozygotes (median ± QD [mg/dL]: 43.00 ± 7.00 vs. 51.00 ± 14.50, respectively; *p* = 0.007) and TC heterozygotes (*p* = 0.022), although the latter difference was significant only before correction for multiple comparisons (Table S1). There was also a difference between T allele carriers and CC homozygotes (median ± QD [mg/dL]: 44.50 ± 8.50 vs. 51.00 ± 14.50, respectively; *p* = 0.002) (Table S2). For the rs13306296 polymorphism, statistically significant differences between GG homozygotes and G allele carriers (consistently higher values of the analyzed parameters) and AA genotypes were observed for CRI-I, CRI-II, and AC indices (Figure 2B). GG homozygotes also displayed higher LDL-C concentrations and higher LCI values; however, these differences lost statistical significance after correction for multiple comparisons (Table S1). Table S1 presents detailed quantitative data (medians ± QD, sample sizes, *p* values) of the analyzed polymorphisms for the additive model, with the exception of rs72811418 (lack of AA homozygotes made it impossible to perform additive analysis) and rs16966671 (lipid parameters did not differ among genotypic variants either before or after correction for multiple comparisons).

Table 3 presents *p*-values from the comparison of serum lipid levels and proatherogenic indices among *CYBA* gene variants in the dominant/recessive model (univariate analysis), while Table S2 provides detailed quantitative data (medians ± QD, sample sizes) for all analyzed polymorphic variants and indices, with the exception of the rs16966671 polymorphism, which also showed no association with the analyzed parameters in the dominant/recessive model.

Next, we examined whether *CYBA* SNPs were associated with lipid parameters in multivariable analysis, after adjusting for age, sex, BMI, smoking status, and hypertension. Analyses were performed for each lipid parameter separately, taking into account all SNPs, separately for the additive and dominant/recessive models. Table 4 presents data for the additive model. Only statistically significant independent predictors are presented. Complete results, including standard errors and t statistics, are provided in Table S3.

The results of the multivariable analysis confirm those obtained in the univariate analysis for rs7195830 and rs1049255 with respect to HDL-C. For rs13306296, in addition to the associations with CRI-I, CRI-II, and AC identified in the univariate analysis, its genotypes were also predictors of TC, LDL-C, and LCI in the multivariable analysis. The AA genotype was associated with a more favorable lipid profile, whereas the GG genotype was associated with a less favorable profile, consistent with the direction of effects observed for CRI-I, CRI-II, and AC. Multivariable regression analysis also indicated that the AA genotype of rs4673 may be a predictor of higher LDL-C levels (Table 4).

An analogous analysis was performed for the dominant/recessive model. In this case, the associations identified in the univariate analysis were also confirmed in the multivariable regression model after adjustment for age, sex, BMI, cigarette smoking, and hypertension (Table 5). In addition to the associations with CRI-I, CRI-II, and AC identified in the univariate analysis, the rs13306296 AA genotype was also a predictor of lower TC and LDL-C levels. Similarly, homozygosity for the TT genotype of rs72811418 was associated with significantly lower CRI-I, CRI-II, and AC values; these comparisons were statistically significant in the univariate analysis before correction for multiple comparisons. Additional associations identified in the multivariable analysis included carriers of the rs4673 A allele with higher TG levels and rs9932581 CC homozygotes with higher LCI values. Complete results for the dominant/recessive model are provided in Table S4.

### 2.4. In Silico Analysis of Allele-Specific Binding for Transcription Factors and Genotype-Related Tissue Expression of CYBA

**Figure 3:**
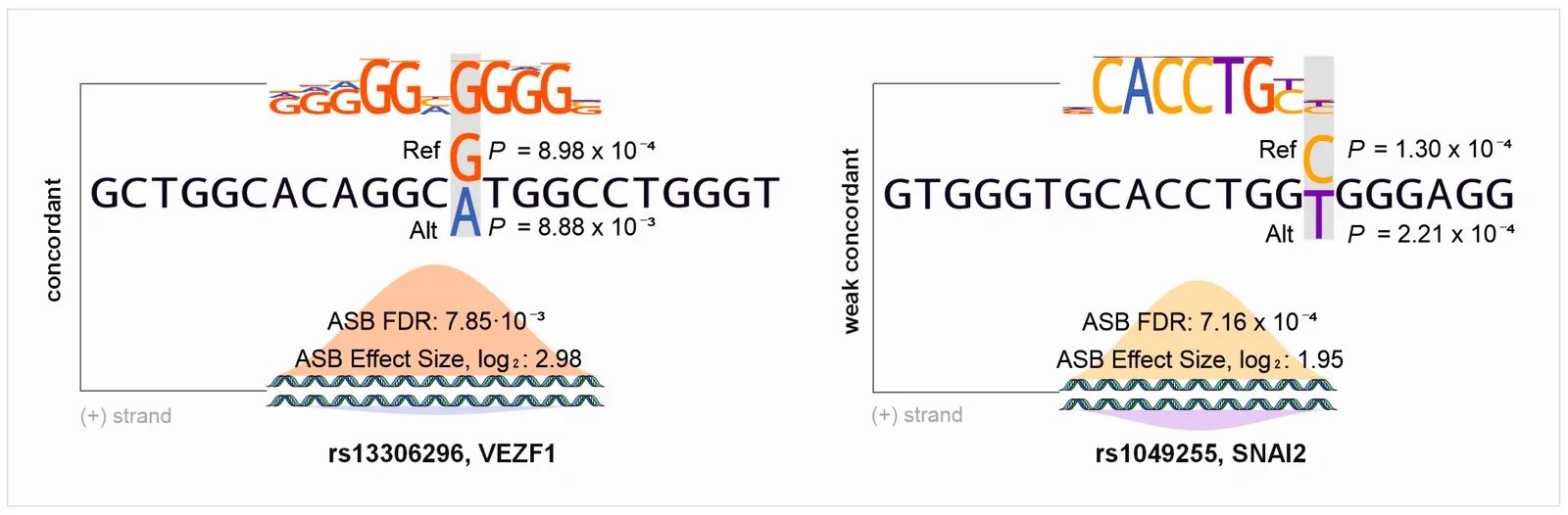
Motif and allele-specific binding effect size illustration of rs13306296 for VEZF1 and rs1049255 for SNAI2. The bells at the bottom of the illustrations correspond to the alleles preferentially bound by the respective transcription factors in vivo. Based on the data from the ADASTRA database [32]. Legend: ASB, Allele-Specific Binding; FDR, False Discovery Rate.

Significant allele-specific binding (ASB) for transcription factors (TFs) was demonstrated for two SNPs: promoter variant rs13306296 and rs1049255 in the 3′ untranslated region. The rs13306296 variant showed ASB for thirteen TFs, with preferential binding associated with the G allele (Table 6), and rs1049255 showed ASB for two TFs (SNAI2, ESR1, with preferential binding associated with the C allele) in K562 (myeloid leukemia) cells. Notably, a corresponding motif fold change, along with motif concordance, was identified only for the VEZF1 (rs13306296) and SNAI2 (rs1049255) transcription factors (Figure 3).

For the other TFs, the observed allele-specific binding could not be directly attributed to a predicted alteration of canonical TF-binding motifs. For the remaining three promoter polymorphisms (rs72811418, rs16966671, and rs9932581) and rs7195830 of the 3′ untranslated region, no ASB events were detected.

Among the seven analyzed polymorphisms, statistically significant differences in *CYBA* gene expression in cardiovascular tissues were observed only between genotypes of two SNPs, namely rs13306296 and rs4673. Homozygotes with the GG genotype of rs13306296 exhibited significantly higher expression levels in the aorta, tibial artery, and heart (left ventricle and atrial appendage) compared to AA homozygotes (Table 7). In the case of the aorta, the results suggest a dominant mode of effect, as elevated *CYBA* expression was observed in both GG homozygotes and GA heterozygotes. In the case of other tissues, the effect of allele dose on the decrease in *CYBA* expression is clearly visible. The findings for the rs4673 polymorphism in the aorta were inconclusive. However, in the tibial artery, AA homozygotes and GA heterozygotes showed higher *CYBA* gene expression compared to GG homozygotes (Table 7).

## 3. Discussion

The present study, whose primary aim was to investigate the potential effects of genotype variants of seven SNPs in the *CYBA* gene on the systemic lipid profile and a panel of lipid-derived atherogenic markers, represents the most comprehensive analysis of this issue to date. The study was conducted in blood donors who had never undergone lipid-lowering therapy and had no clinical signs of cardiovascular disease. In the course of the study, we demonstrated statistically significant differences in lipid parameters among the genotypes of four of the polymorphisms investigated, namely rs7195830, rs1049255, rs72811418, and rs13306296. In silico analyses also indicated the possibility of allele-specific binding of transcription factors to two of the above variants (rs1049255 and rs13306296), as well as differential, genotype-dependent effects of some polymorphisms (rs13306296 and rs4673) on *CYBA* gene expression.

The promoter variant rs13306296 (historical designation: −536 C>T) stands out among the SNPs analyzed. In the present study, the GG genotype/G allele (depending on the genetic model applied) showed multiple associations with lipid parameters and atherogenic indices. GG homozygotes had higher CRI-I, CRI-II, and AC values than AA homozygotes, as well as higher TC, LDL-C and LCI levels in multivariable analysis than AA homozygotes. The distribution of medians (Figure 2B, Table S1) suggests an additive effect, with progressively higher values of lipids/atherogenic indices observed with an increasing number of G alleles in the genotype. In the dominant/recessive model, higher values of TC, LDL-C and the same indices were observed in G-allele carriers (GG/GA genotypes) compared with AA homozygotes. In silico analyses indicated that the presence of the G allele in the promoter may account for preferential binding of thirteen transcription factors. However, the absence of motif concordance, despite significant allele-specific binding for the G allele, was observed for twelve TFs. This may suggest the involvement of mechanisms other than simple alterations of canonical DNA sequences, such as indirect recruitment through protein–protein interactions or effects mediated by the local chromatin context. With regard to the effect of rs13306296 on gene expression, the G allele was associated with increased *CYBA* expression in the aorta under a dominant model, whereas in the tibial artery and cardiac tissues, the observed effect was additive, with the highest expression in GG homozygotes and the lowest in AA homozygotes.

Despite the clear impact of rs13306296 on the lipid profile and *CYBA* expression observed in GTEx data, this variant remains one of the least investigated polymorphisms of the gene, and the available evidence is scarce. According to the literature, rs13306296 has not been associated with hypertension [34], CAD [35], or coal workers’ pneumoconiosis [36]. In our previous study, although the GG genotype of this polymorphism was not associated with an increased risk of CAD, it was more frequent among patients with peripheral vascular disease [35]. Based on these limited data, it appears that rs13306296 may influence gene expression in cardiovascular tissues and, consequently, lipid-related indices, although these effects appear to translate only to a limited extent into the clinical phenotype.

The analysis of transcription factors that showed preferential binding to the G allele of rs13306296 in the in silico analysis does not provide additional insight. Although many of these TFs are involved in adipogenesis, lipid metabolism, and free-radical homeostasis, the extent to which they regulate *CYBA* expression remains unclear, and the functional consequences of TF binding at this locus remain to be experimentally validated in cardiovascular tissues. The principal functions of these TFs relevant to the subject of the present study are summarized in Table 8, which we have included solely out of authorial obligation.

The remaining polymorphisms in the promoter region, namely rs72811418 (−675 A>T), rs16966671 (−852 C>G), and rs9932581 (−930 A>G), showed no associations with lipid profiles in univariate analysis, showed no allele-specific TF binding, and had no effect on *CYBA* gene expression in the in silico analyses. However, the results concerning the rs72811418 variant obtained in multivariable regression analysis are noteworthy, as AT heterozygotes had significantly lower HDL-C concentrations and significantly higher values of the pro-atherogenic indices CRI-I, CRI-II, and AC compared with TT homozygotes (Table S2). According to Moreno et al. [34], the rs72811418 polymorphism is located within a potential binding site for the HIF-1α (hypoxia-inducible factor-1α) transcription factor. Substitution of A with T leads to the loss of the HIF-1α binding sequence, and the A allele was found to be associated with p22phox overexpression in response to HIF-1α, increased NADPH oxidase activity and superoxide anion production, hypertension, and greater carotid artery intima-media thickness [34]. In our previous studies, the T allele of rs72811418, when present in a diplotype with the T allele of rs9932581, was associated with a lower risk of CAD in women [35]. Moreover, TT homozygosity was associated with better 5- and 10-year survival among patients with coronary artery disease [62], which, in the context of the findings of the present study, does not appear to be coincidental.

The 3′ UTR polymorphisms of the *CYBA* gene, namely rs7195830 (*49 A>G) and rs1049255 (*24 A>G, A640G), were also associated with HDL levels in both the additive model (both in univariate and multivariable analysis) and dominant/recessive model (only in univariate model). Carriers of the G allele (rs7195830) and the T allele (rs1049255) were characterized by a less favorable lipid profile. In addition, rs1049255 showed ASB for two TFs (SNAI2 and ESR1), with preferential binding of both TFs to the C allele. The effects of both polymorphisms on *CYBA* expression remain unclear in the literature. Fan et al. [63] suggested that rs7195830 is located within a miRNA-binding region. Since miRNA binding to mRNA is essential for the regulation of mRNA levels and protein expression, it is possible that allelic variants of rs7195830 may differentially affect the efficiency and kinetics of these processes through miRNA-mediated mechanisms. Previous in silico studies have indeed suggested that the rs7195830 genotype may be associated with differences in *CYBA* expression, for example, in the small intestine [64]; however, this does not apply to the tissues of the vascular system. In our previous studies, carriage of the G allele of rs7195830 was a risk factor for CAD [65], whereas the presence of the A allele in a diplotype with the T allele of rs9932581 reduced the risk of CAD in women [35]. In light of the findings of the present study, the protective effect of the rs7195830 A allele may be attributable to its favorable effect on HDL-C levels and lipid indices. In the case of the other 3′ UTR polymorphism, rs1049255, previous studies in healthy volunteers showed that the C allele was associated with lower mRNA levels, lower protein expression, and lower NADPH oxidase activity in peripheral blood granulocytes [66]. It is therefore possible that the higher HDL-C levels observed in CC homozygotes in the present study may be related to reduced *CYBA* expression in granulocytes and, consequently, lower free-radical production. However, it should be noted that the currently available data from the GTEx Portal (for arteries, heart, and whole blood) do not support this hypothesis. The database still lacks gene-expression data for individual blood cell types. In this context, data from neutrophils and macrophages would be particularly informative.

The last polymorphism investigated was the exonic rs4673 (214 C>T, C242T), the most extensively studied SNP in the *CYBA* gene. According to the results of meta-analyses, carriage of the A allele/homozygosity for AA (depending on the genetic model applied) is associated with an increased risk of CAD and acute cardiovascular events in Caucasian populations, whereas GG homozygosity is protective [18,19,21,22]. Similarly, in our previous studies, carriage of the A allele of rs4673 showed a synergistic association with smoking and hypercholesterolemia, markedly increasing the risk of CAD [67]. In the current study, rs4673 was a rather weak predictor of lipid parameters, with AA homozygosity being associated with elevated LDL-C levels and A allele carriage (AA/GA) with higher TG levels. The tendency toward a more favorable lipid profile among GG homozygotes observed in our study is supported by the literature. In a Russian population, AA homozygotes had significantly higher TC and LDL-C levels, whereas GG homozygotes had non-significantly higher HDL-C levels, both among patients with CAD and in the control group [68]. Similarly, studies in a cohort of Polish patients with type 2 diabetes mellitus revealed the highest TC, TG, and LDL-C levels among AA homozygotes, although statistically significant differences were observed only for TC [69]. Returning to the findings of the present study, the in silico analysis also demonstrated an effect of rs4673 on *CYBA* expression in the aorta and tibial artery, with the highest gene expression observed in heterozygotes. Taken together, these findings suggest that rs4673 should be considered in future studies investigating the effects of genetic variation in *CYBA* on human lipid metabolism.

The findings of the present study, together with the available literature data, indicate that polymorphisms in the *CYBA* gene may modulate systemic lipid homeostasis through their effects on *CYBA* gene expression and, consequently, on the activity of vascular NADPH oxidases. The effects of free radicals on both LDL-C oxidation and the increased expression of genes regulating lipo- and cholesterologenesis appear, in theory, to be relatively clear and have been supported by numerous studies [7,10,13,14,25,26,27,28,29], and may account for the differences in atherogenic indices between *CYBA* variants observed in the present study. The effect of free radicals on HDL-C levels represents a separate issue. Serum HDL-C concentration depends on a complex interplay of genetic factors, lifestyle (physical activity, diet, smoking, BMI), and the metabolic and hormonal status of the organism [70,71]. Apart from traditional modifiable factors, key biological determinants include ATP-binding cassette subfamily A member 1 (ABCA1), apolipoprotein A-I (Apo A-I), lecithin–cholesterol acyltransferase (LCAT), cholesteryl ester transfer protein (CETP), as well as the scavenger receptor class B type 1 (SR-B1), all of which are involved in reverse cholesterol transport (RCT) from peripheral tissues to the liver [72,73]. Cholesterol from peripheral tissue cells is initially transferred by the ABCA1 transporter, a membrane protein of the ABC family responsible for cholesterol efflux from cells, to Apo A-I, resulting in the formation of nascent, discoidal pre-β-HDL particles. Apo A-I, the major protein component of HDL, is responsible both for interactions with ABCA1 transporters and for the direct activation of LCAT, an enzyme that esterifies free cholesterol in the bloodstream by transferring a fatty acid residue from lecithin to cholesterol. This process promotes HDL maturation, resulting in the formation of spherical particles that can accept additional cholesterol with the involvement of the membrane proteins ABCG1 (ATP-binding cassette subfamily G member 1) and ABCG4. CETP, in turn, transfers cholesteryl esters (CE) from HDL to VLDL/LDL in exchange for triglycerides, and its high activity reduces HDL-C levels. Mature HDL delivers cholesteryl esters to hepatocytes via the SR-B1 receptor, which enables selective lipid transfer without HDL endocytosis. Cholesterol in the liver may subsequently be used for bile acid synthesis, secreted into bile, or utilized for the formation of cell membranes and very-low-density lipoproteins (VLDL) [72,73].

**Figure 4:**
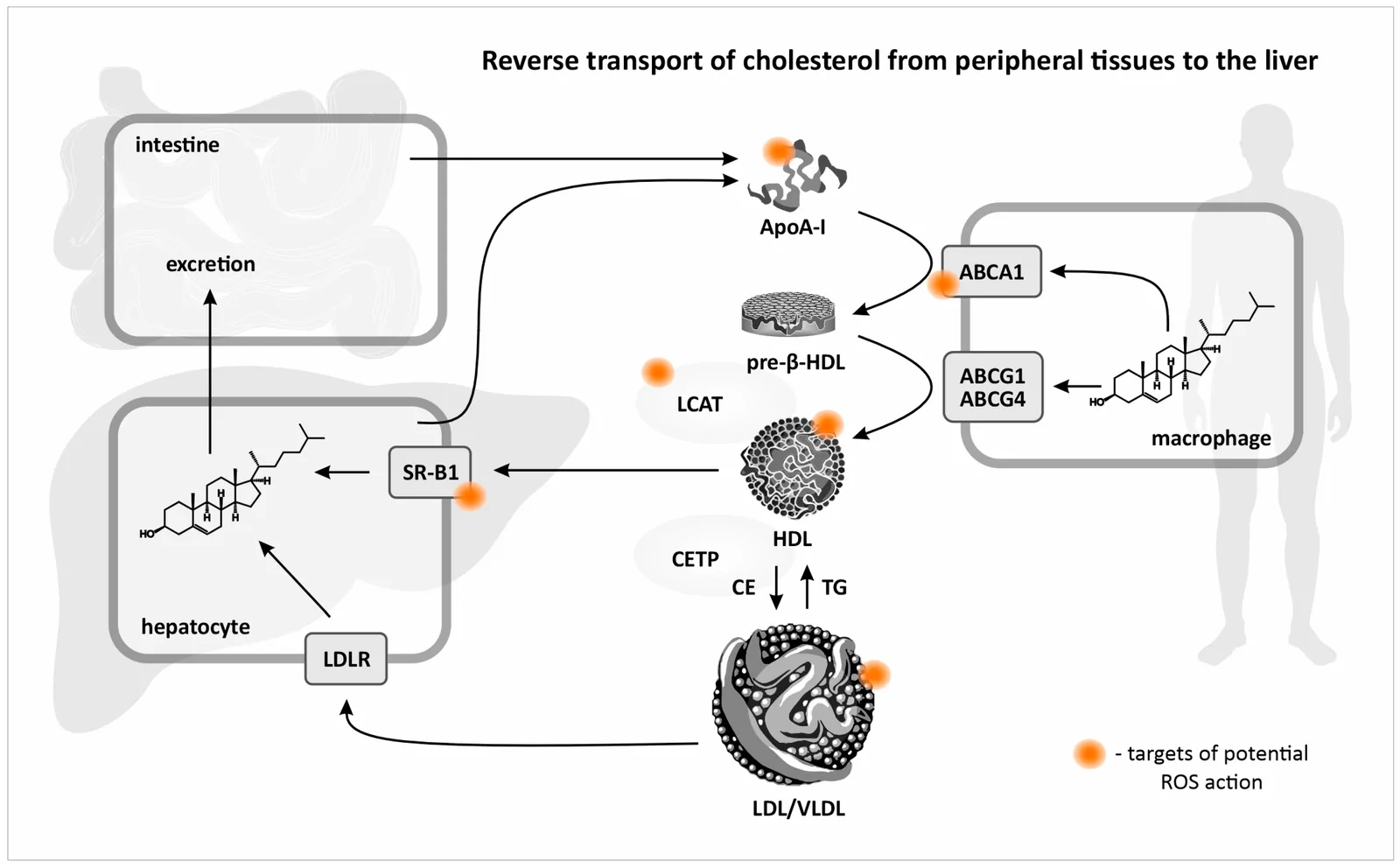
Targets of potential ROS action in molecules involved in reverse cholesterol transport from peripheral tissues to the liver (description in the text). Pictures of HDL and LDL molecules based on figures from the Servier Medical Art resource [82], licensed under CC BY 4.0. (modified).

The effect of oxidative stress on reverse cholesterol transport is generally inhibitory and involves multiple mechanisms. Components of the RCT pathway that appear to be particularly susceptible to free-radical-mediated damage include ABCA1, Apo A-I, HDL, and LCAT (Figure 4). Both direct oxidation of ABCA1 and lipid peroxidation of cellular membranes lead to reduced protein activity, while ROS-induced membrane stiffening decreases its mobility and efficiency in cholesterol uptake within lipid rafts [74,75,76]. This results in cholesterol retention within cells and reduced serum HDL levels, the formation of foam cells that contribute cholesterol deposits to the developing atherosclerotic plaque, and a local increase in inflammation [76,77,78]. Although HDL oxidation is less well understood than LDL oxidation, available data suggest that oxHDL is generated under the influence of hypochlorous acid produced by macrophage myeloperoxidase from chloride ions and H_2_O_2_, the latter being generated from the superoxide anion produced by NADPH oxidases [75,79,80]. New data also indicate that HDL and Apo A-I oxidation may result from exposure to the superoxide anion, while Apo A-I may be oxidized either directly by the superoxide anion or indirectly by HDL lipids previously oxidized by this radical [81]. Regardless of the oxidant involved, Apo A-I oxidation leads to structural changes, formation of high-molecular-weight adducts between Apo A-I molecules as well as with other HDL apoproteins and lipids, and consequently to loss of protein function and the anti-atherogenic properties of HDL [80,81].

However, there is no direct relationship between oxidized HDL and serum HDL-C levels; moreover, there is evidence of a correlation between the concentrations of these two parameters [83], while HDL-C levels do not provide information about HDL functionality or its oxidative modifications [84,85]. Regarding LCAT, its activity decreases markedly under experimental conditions following exposure to both the superoxide anion [81] and hydrogen peroxide [86]; however, in this case as well, there is no direct evidence of a reduction in HDL-C concentration in response to oxidative stress. A likely explanation for this may be the methodology used by commercial colorimetric assays for HDL-C determination, including the assay used in the present study, which is based on the detection of free cholesterol following prior hydrolysis of cholesteryl esters by cholesterol esterase. Consequently, the pool of cholesterol detected in HDL may be independent of LCAT activity and cholesteryl ester concentration, as the entire cholesterol pool will ultimately be detected in the subsequent assay reactions regardless of LCAT activity. SR-B1, the membrane cholesterol transporter of hepatocytes and the final component of the RCT pathway, may also represent a potential target of oxidation. In a mouse model, physiological levels of H_2_O_2_ were shown to increase SR-B1-mediated cholesteryl ester uptake by 65% [87]. The situation may change under oxidative stress, during which oxidized HDL/Apo A-I loses its ability to bind to SR-B1 [88]. In summary, there is limited evidence linking oxidative stress to reduced HDL-C levels, with the strongest evidence concerning oxidation of ABCA1 and Apo A-I/HDL; however, in the latter case, the observed effects are multifaceted and largely dependent on the biological context. The involvement of NADPH oxidases in these processes, and even more so the influence of *CYBA* gene polymorphisms, remains inferential, although these enzymes are undoubtedly major sources of superoxide anion in tissues of the vascular system, and some *CYBA* polymorphisms modulate gene expression. Further studies are clearly needed to investigate the effects of NADPH oxidase activity on lipid metabolism, HDL dysfunction, and reverse cholesterol transport. The effects of *CYBA* gene polymorphisms on gene expression in neutrophils and macrophages also require clarification, as does the influence of promoter SNP variants on transcription factor binding in cardiovascular tissues and leukocytes. Such knowledge may contribute to a better understanding of the pathogenesis of atherosclerosis and provide a basis for the development of novel therapies. Although the multifaceted contribution of NADPH oxidase-derived free radicals to the development of cardiovascular diseases has been demonstrated over the years, the findings of the present study suggest that their effects on systemic lipid homeostasis may also represent an important aspect of their biological activity.

A limitation of the present study is the relatively small study population, which may have resulted in an underestimation of the observed effects of SNP variants on lipid parameters. Another limitation is the selective nature of the results derived from the in silico analyses. ADASTRA data were obtained from K562 cells (myelogenous leukemia cells) and should therefore be interpreted with caution; however, we consider them supplementary data that do not affect the results of the present study. A final important limitation of the present study is the lack of established genome-wide significant associations between the investigated *CYBA* SNPs and lipid traits in the GWAS Catalog. To further evaluate our findings, we examined the full summary statistics from the large multi-ancestry GLGC (Global Lipids Genetics Consortium) meta-analysis reported by Graham et al. [89], which included approximately 1.65 million participants from five ancestry groups. All seven *CYBA* variants investigated in our study were represented in the GLGC dataset, and several showed associations with lipid traits, although none reached the conventional genome-wide significance threshold (*p* < 5 × 10^−8^). Importantly, the direction of effects was concordant with the significant genotype-dependent differences observed in our study, with particularly notable consistency for rs4673 and rs13306296 (Table S5). Nevertheless, direct comparison between the studies is limited by substantial differences in design. The GLGC meta-analysis combined 201 studies from 35 countries and multiple ancestry groups, resulting in considerable population and clinical heterogeneity, and individual-level information on lipid-lowering therapy is not available in the analyzed summary statistics. In contrast, our cohort was geographically and ethnically homogeneous, and participants reported no use of lipid-lowering medications. Furthermore, most of our significant findings concerned composite atherogenic indices rather than individual lipid fractions, whereas such indices were not directly assessed in the GLGC analysis. Therefore, our findings should be considered candidate-gene associations supported by directionally concordant large-scale data but requiring further independent replication.

In conclusion, *CYBA* gene polymorphisms, especially rs13306296, rs7195830, and rs1049255, affect systemic lipid status and a panel of lipid-derived atherogenic markers in individuals who had never undergone lipid-lowering therapy.

## 4. Materials and Methods

### 4.1. Study Design

This cohort study of Polish blood donors, who had never undergone lipid-lowering therapy, was conducted in accordance with STROBE guidelines. Seven single nucleotide polymorphisms of the *CYBA* gene were genotyped, and serum lipid levels, as well as values of lipid-derived atherogenic indices, were compared between genotypic variants of all SNPs in the additive and dominant/recessive modes. Additionally, the potential impact of *CYBA* gene polymorphisms on gene expression was assessed in silico in cardiovascular tissues.

### 4.2. Subjects

The study group consisted of 220 blood donors (67 women, 153 men) with a median age of 44.00 ± 4.50 years. Participants were recruited from the Regional Centers for Blood Donation and Blood Treatment in Katowice and Racibórz as a control group for a case–control study on the genetic factors of coronary artery disease (CAD) in Poland. All subjects were Polish Caucasians and residents of Upper Silesia (southern Poland). Exclusion criteria included symptoms of CAD, myocardial infarction (MI), stroke, diabetes mellitus, inflammatory or autoimmune diseases, and a family history of cardiovascular diseases. The presence of cardiovascular or other diseases was ruled out based on a physical examination and a medical interview. A family history was defined as CAD, MI, or stroke occurring in at least one parent. In compliance with the nationwide recommendations of Polish Centers of Blood Donation and Blood Treatment, the blood samples were obtained only from patients with systolic blood pressure less than 140 mmHg and diastolic blood pressure less than 90 mmHg on the day of blood collection. Subjects included in the study did not report receiving lipid-lowering agent treatment. Blood samples were collected between 2001 and 2013, while DNA isolation and genotyping were performed between 2001 and 2018. The study was conducted in accordance with the Declaration of Helsinki. Written informed consent was obtained from each participant, and the study protocol was approved by the Bioethics Committee of the Medical University of Silesia in Katowice.

### 4.3. Genetic Analyses

**Figure 5:**
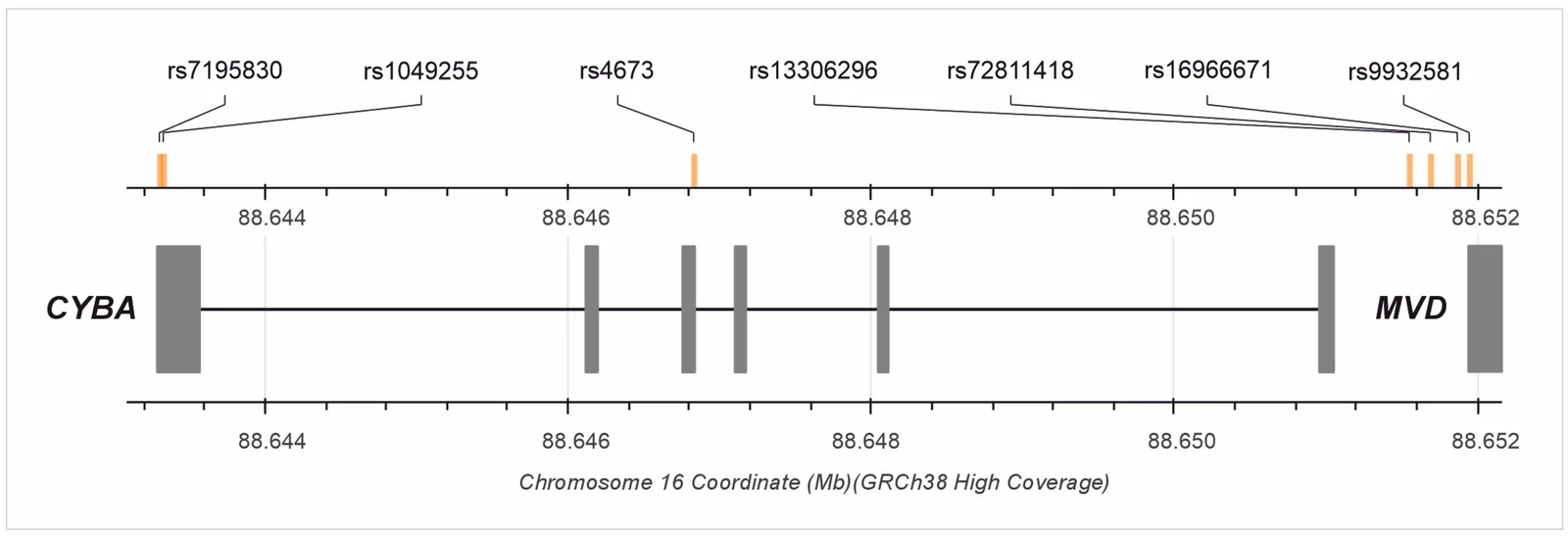
Localization of *CYBA* gene polymorphisms within the gene, based on data from LDmatrix Tool [31].

DNA was isolated from fresh blood samples using the MasterPure Genomic DNA Purification Kit (Epicentre Technologies, Madison, WI, USA). Seven *CYBA* gene polymorphisms were analyzed: rs7195830 (1), rs1049255 (2), rs4673 (3), rs13306296 (4), rs72811418 (5), rs16966671 (6), and rs9932581 (7). The selection of SNPs for the study was based on their location within regulatory regions of the gene (promoter, 3′ UTR). The exonic variant rs4673 was an exception. It was included in the study due to numerous associations with atherosclerosis-related phenotypes reported in the literature. The locations of the analyzed SNPs in the *CYBA* gene are shown in Figure 5.

Polymorphisms 1 and 7 were genotyped using the TaqMan Predesigned SNP Genotyping Assay and the 7300 Real-Time PCR System (Applied Biosystems, Foster City, CA, USA). The remaining SNPs were genotyped using the PCR-RFLP method as described by Inoue et al. [90] (for polymorphisms 2 and 3) and Moreno et al. [34] (for SNPs 4–6). In the case of PCR-RFLP analysis, PCR amplifications were performed using a T-GRADIENT thermocycler (Biometra, Göttingen, Germany). To verify genotyping accuracy, 10–15% of the samples were regenotyped for each polymorphism. The repeatability was 100%.

### 4.4. Biochemical Analyses

All examined individuals were instructed to fast for at least 14 h before blood collection. Venous blood was collected, and samples were centrifuged within 2 h of being drawn. TC, HDL-C and TG were measured in fresh serum by commercial enzymatic methods (Analco, Warszawa, Poland) twice for each subject. If the results differed by 10% or more, an additional third measurement was performed. LDL-C levels were calculated using the Friedewald formula [91] in participants with TG levels below 4.4 mmol/L.

Values of six lipid-derived atherogenic indices, namely Castelli’s risk index I (CRI-I), Castelli’s risk index II (CRI-II) [92], the lipid combination index (LCI) [93], the atherogenic coefficient (AC) [94], the atherogenic index of plasma (AIP) [95], and the triglyceride-to-HDL-C ratio (TG/HDL-C) [96], were calculated using the following formulas (Table 9). Serum lipid concentrations and the values of atherosclerotic indices were compared between genotypic variants of individual *CYBA* gene polymorphisms.

### 4.5. In Silico Analyses

For each analyzed SNP, in silico analysis of allele-specific binding (ASB) for transcription factors (based on the data from the ADASTRA, Allelic Dosage-corrected Allele-Specific human Transcription factor binding sites database) [32,97,98] and analysis of expression quantitative trait loci (based on the data obtained from the GTEx, The Genotype-Tissue Expression Portal) [33] were performed to determine whether the studied polymorphisms affect *CYBA* expression. Expression in arteries (aorta, coronary arteries, tibial artery), heart tissues (left ventricle, atrial appendage), and whole blood was analyzed.

### 4.6. Statistical Analyses

Data were analyzed using Statistica 13.0 software (TIBCO Software Inc, Palo Alto, CA, USA). The normality of the quantitative data distribution was computed using the Shapiro–Wilk test. All quantitative variables exhibited a non-normal distribution, so the results were reported as medians (±QD, quartile deviation). The Mann–Whitney U test (for dichotomous grouping variables) and the Kruskal–Wallis test (for grouping variables with more than two categories) were used to compare quantitative variables. Spearman’s rank correlation coefficient (rs) was used as a measure of the correlation between quantitative variables. Cases with missing data were excluded from the relevant comparisons. Genetic data were analyzed under additive and dominant/recessive inheritance models. The *χ*^2^ test was used to assess Hardy–Weinberg equilibrium, as well as to compare genotype variant frequencies across categories of qualitative variables (sex, overweight/obesity, cigarette smoking). Yates’ correction was applied to subgroups with fewer than ten subjects. Haplotype analysis was performed using HaploView 4.2. software [99] and the algorithm by Gabriel et al. [100]. The D’ and R^2^ values were used as measures of linkage disequilibrium. Statistical significance was set at *p* < 0.050 in univariate analysis, and the Bonferroni–Hochberg correction for multiple comparisons was used in univariate analysis [101]. The correction was calculated for all SNPs (separately for the additive and dominant/recessive models), taking into account age and sex, comorbidities (overweight/obesity and hypertension) and cigarette smoking. Sample size and power analysis were performed, and the sample size of the study group was sufficient to obtain 99% power (95% two-sided confidence level) in all statistically significant tests after Bonferroni–Hochberg correction for multiple comparisons. Values of B (adjusted unstandardized regression coefficient) and β (standardized regression coefficient), as well as their 95% CIs, were computed using multivariable linear regression analysis after adjustment for age, sex, BMI, cigarette smoking status and hypertension.

### 4.7. Using GenAI

Generative artificial intelligence (GenAI) was used to edit the text for grammar, spelling, and punctuation.

## Figures and Tables

**Table 1:** General and biochemical characteristics of the study group.

Characteristics		
Number of subjects, n %	220	100.00
Age [years], median ± QD	44.00	4.50
Males, n %	153	69.55
Females, n %	67	30.45
BMI [kg/m^2^], median ± QD	26.00	2.60
BMI ≥ 25, n %	132	60.00
BMI ≥ 30, n %	32	14.55
TC [mg/dL], median ± QD	202.00	29.00
HDL-C [mg/dL], median ± QD	46.00	9.00
LDL-C [mg/dL], median ± QD	126.00	28.90
TG [mg/dL], median ± QD	124.00	44.00
CRI-I [mg/dL], median ± QD	4.46	1.05
CRI-II [mg/dL], median ± QD	2.80	0.91
LCI [mmol/L], median ± QD	22.66	14.90
AC [mmol/L], median ± QD	3.46	1.05
AIP [mg/dL], median ± QD	0.44	0.20
TG/HDL-C [mg/dL], median ± QD	2.77	1.25
Hypertension, n %	4	1.82
Cigarette smoking, n %	58	26.36

Legend: AC, atherogenic coefficient; AIP, atherogenic index of plasma; BMI, body mass index; CRI-I, Castelli’s risk index I; CRI-II, Castelli’s risk index II; HDL-C, high-density lipoprotein cholesterol; LCI, lipid combination index; LDL-C, low-density lipoprotein cholesterol; TC, total cholesterol; TG, triacylglycerols; TG/HDL-C, triglyceride-to-HDL-C ratio; QD, quartile deviation.

**Table 2:** The frequency of genotypes and alleles of analyzed SNPs of the *CYBA* gene.

SNP *	GRCh38 Coordinate	Genotypes	n, %	Alleles	n, %	MAF in Europe **	*p-*Value HWE Test
rs7195830	chr16:88643304	AA	51, 23.72	A	201, 47.18	44.64	0.548
(*49 A>G)		AG	99, 46.05	G	225, 52.82		
		GG	65, 30.23				
rs1049255	chr16:88643329	TT	64, 29.09	T	234, 53.18		0.891
(*24 A>G,		TC	106, 48.18	C	206, 46.82	48.48	
A640G)		CC	50, 22.73				
rs4673	chr16:88646828	GG	94, 44.13	G	279, 65.49		0.704
(214 C>T,		AG	91, 42.72	A	147, 34.51	34.30	
C242T)		AA	28, 13.15				
rs13306296	chr16:88651549	GG	93, 43.87	G	280, 66.04		0.989
(−536 C>T)		GA	94, 44.34	A	144, 33.96	35.03	
		AA	25, 11.79				
rs72811418	chr16:88651688	AA	0	A	28, 6.48	7.44	0.424
(−675 A>T)		AT	28, 12.96	T	404, 93.52		
		TT	188, 87.04				
rs16966671	chr16:88651867	GG	45, 21.13	G	197, 46.24	47.26	0.987
(−852 C>G)		GC	107, 50.23	C	229, 53.76		
		CC	61, 28.64				
rs9932581	chr16:88651945	TT	41, 18.81	T	186, 42.66	40.80	0.921
(–930 A>G)		TC	104, 47.71	C	250, 57.34		
		CC	73, 33.49				

Legend: GRCh38, Genome Reference Consortium Human Build 38 Organism: Homo sapiens; HWE, Hardy–Weinberg equilibrium; MAF, minor allele frequency; SNP, single nucleotide polymorphism; *, rs number (reference SNP-cluster ID) and historical name used in the literature (in brackets); **, data from the ALFA Allele Frequency for European population [30].

**Table 3:** *p*-values from the comparison of serum lipid levels and proatherogenic indices among *CYBA* gene variants in the dominant/recessive model (univariate analysis).

SNP	Comparison	*p* Value, Mann–Whitney U Test								
		**HDL-C**	**LDL-C**	**TG**	**CRI-I**	**CRI-II**	**LCI**	**AC**	**AIP**	**TG/HDL-C**
rs7195830	GG/AG ^§^ vs. AA	<0.001*	0.847	0.037	0.009	0.029	0.025	0.009	0.001 *	0.001 *
rs1049255	TT/TC ^§^ vs. CC	0.002 *	0.172	0.646	0.019	0.020	0.157	0.019	0.078	0.078
rs4673	AA/AG ^§^ vs. GG	0.027	0.648	0.561	0.083	0.073	0.399	0.083	0.180	0.180
rs13306296	GG/GA ^§^ vs. AA	0.080	0.010	0.270	0.004 *	0.001 *	0.021	0.004 *	0.107	0.107
rs72811418	AT ^§^ vs. TT	0.020	0.271	0.460	0.022	0.018	0.085	0.022	0.074	0.074
rs9932581	CC ^§^ vs. TC/TT	0.063	0.168	0.238	0.067	0.074	0.077	0.067	0.032	0.032

Legend: AC, atherogenic coefficient; AIP, atherogenic index of plasma; CRI-I, Castelli’s risk index I; CRI-II, Castelli’s risk index II; HDL-C, high-density lipoprotein; LCI, lipid combination index; LDL-C, low-density lipoprotein; SNP, single nucleotide polymorphism; TC, total cholesterol; TG, triglycerides; TG/HDL-C, triglyceride-to-HDL-C ratio; ^§^, the risk variant with lower HDL-C levels and/or higher levels of other parameters; *, differences remaining significant after Bonferroni–Hochberg correction for multiple comparisons (threshold of significance for the dominant/recessive model: *p* ≤ 0.004).

**Table 4:** Predictors of lipid parameters in the study group in the additive model (multivariable analysis after adjustment for age, sex, BMI, cigarette smoking status, and hypertension).

Lipid Parameter	Predictor	Variant	B	95% CI	β	95% CI	*p*
TC [mg/dL]	rs13306296	GG	16.11	(4.40–27.81)	0.34	(0.09–0.59)	0.007
	rs13306296	AA	−18.32	(−33.31–−3.33)	−0.27	(−0.49–−0.05)	0.017
	Age		1.31	(0.30–2.31)	0.19	(0.04–0.33)	0.011
	BMI		1.98	(0.33–3.63)	0.18	(0.03–0.33)	0.019
HDL-C	rs7195830	AA	7.40	(1.63–13.18)	0.40	(0.09–0.71)	0.012
[mg/dL]	rs1049255	TT	−5.99	(−11.77–−0.22)	−0.32	(−0.63–−0.01)	0.042
LDL-C	rs13306296	GG	17.47	(6.54–28.39)	0.40	(0.15–0.65)	0.002
[mg/dL]	rs13306296	AA	−22.97	(−36.94–−9.00)	−0.36	(−0.59–−0.14)	0.001
	Age		1.26	(0.32–2.20)	0.20	(0.05–0.34)	0.009
	rs4673	AA	11.06	(0.28–21.85)	0.18	(0.00–0.36)	0.044
TG [mg/dL]	BMI		5.95	(2.86–9.05)	0.28	(0.14–0.43)	<0.001
CRI-I [mg/dL]	rs13306296	AA	−0.75	(−1.33–−0.16)	−0.28	(−0.50–−0.06)	0.012
	Age		0.06	(0.02–0.10)	0.22	(0.08–0.37)	0.003
CRI-II [mg/dL]	rs13306296	AA	−0.69	(−1.19–−0.19)	−0.31	(−0.53–−0.09)	0.007
	Age		0.05	(0.01–0.08)	0.21	(0.06–0.35)	0.005
LCI [mmol/L]	rs13306296	GG	10.74	(1.69–19.79)	0.29	(0.05–0.54)	0.020
	rs13306296	AA	−11.98	(−23.53–−0.43)	−0.23	(−0.45–−0.01)	0.042
	Age		1.23	(0.44–2.02)	0.22	(0.08–0.37)	0.002
	BMI		1.70	(0.43–2.97)	0.20	(0.05–0.34)	0.009
AC [mmol/L]	rs13306296	AA	−0.75	(−1.33–−0.16)	−0.28	(−0.50–−0.06)	0.012
	Age		0.06	(0.02–0.10)	0.22	(0.08–0.37)	0.003
AIP [mg/dL]	BMI		0.02	(0.01–0.03)	0.23	(0.08–0.38)	0.002
	Age		0.01	(0.00–0.02)	0.18	(0.04–0.33)	0.011
TG/HDL-C	BMI		0.12	(0.04–0.21)	0.21	(0.06–0.36)	0.006
[mg/dL]	Age		0.07	(0.01–0.12)	0.18	(0.04–0.33)	0.015

Legend: B, adjusted unstandardized regression coefficient (effect in lipid parameter units); β, standardized regression coefficient (to compare the strength of individual predictors); CI, confidence interval. AC, atherogenic coefficient; AIP, atherogenic index of plasma; BMI, body mass index; CRI-I, Castelli’s risk index I; CRI-II, Castelli’s risk index II; HDL-C, high-density lipoprotein cholesterol; LCI, lipid combination index; LDL-C, low-density lipoprotein cholesterol; TC, total cholesterol; TG, triacylglycerols; TG/HDL-C, triglyceride-to-HDL-C ratio.

**Table 5:** Predictors of lipid parameters in the study group in the dominant/recessive model; multivariable regression analysis after adjustment for age, sex, BMI, cigarette smoking, and hypertension.

Lipid Parameter	Predictor	Variant	B	95% CI	β	95% CI	*p*
TC [mg/dL]	BMI		2.13	(0.50–3.76)	0.19	(0.05–0.34)	0.011
	rs13306296	AA	−13.79	(−24.83–−2.76)	−0.19	(−0.34–−0.04)	0.015
	Age		1.26	(0.26–2.27)	0.18	(0.04–0.33)	0.014
LDL-C [mg/dL]	rs13306296	AA	−17.12	(−27.35–−6.89)	−0.25	(−0.41–−0.10)	0.001
	Age		1.21	(0.27–2.14)	0.19	(0.04–0.33)	0.012
TG [mg/dL]	BMI		6.45	(3.42–9.48)	0.31	(0.16–0.45)	<0.001
	rs4673	AA/GA	13.93	(0.12–27.74)	0.16	(0.00–0.33)	0.048
CRI-I [mg/dL]	Age		0.06	(0.02–0.10)	0.22	(0.08–0.36)	0.003
	rs72811418	TT	−0.62	(−1.05–−0.19)	−0.22	(−0.38–−0.07)	0.005
	rs13306296	AA	−0.52	(−0.95–−0.09)	−0.18	(−0.34–−0.03)	0.018
CRI-II [mg/dL]	rs72811418	TT	−0.56	(−0.92–−0.19)	−0.24	(−0.39–−0.08)	0.003
	rs13306296	AA	−0.50	(−0.86–−0.13)	−0.21	(−0.36–−0.06)	0.008
	Age		0.05	(0.01–0.08)	0.20	(0.06–0.35)	0.006
LCI [mmol/L]	Age		1.18	(0.40–1.97)	0.22	(0.07–0.36)	0.003
	BMI		1.92	(0.67–3.16)	0.22	(0.08–0.37)	0.003
	rs9932581	CC	6.11	(0.84–11.37)	0.17	(0.02–0.32)	0.023
AC [mmol/L]	Age		0.06	(0.02–0.10)	0.22	(0.08–0.36)	0.003
	rs72811418	TT	−0.62	(−1.05–−0.19)	−0.22	(−0.38–−0.07)	0.005
	rs13306296	AA	−0.52	(−0.95–−0.09)	−0.18	(−0.34–−0.03)	0.018
AIP [mg/dL]	Age		0.01	(0.00–0.02)	0.17	(0.03–0.32)	0.018
	BMI		0.02	(0.01–0.03)	0.25	(0.11–0.40)	0.001
TG/HDL-C [mg/dL]	Age		0.06	(0.01–0.12)	0.17	(0.03–0.32)	0.021
	BMI		0.14	(0.05–0.22)	0.23	(0.08–0.38)	0.003

Legend: B, adjusted unstandardized regression coefficient (effect in lipid parameter units); β, standardized regression coefficient (to compare the strength of individual predictors); CI, confidence interval. AC, atherogenic coefficient; AIP, atherogenic index of plasma; BMI, body mass index; CRI-I, Castelli’s risk index I; CRI-II, Castelli’s risk index II; LCI, lipid combination index; LDL-C, low-density lipoprotein cholesterol; TC, total cholesterol; TG, triacylglycerols; TG/HDL-C, triglyceride-to-HDL-C ratio.

**Table 6:** Allele-specific binding for transcription factors with respect to the rs13306296 alleles, based on the data from the ADASTRA (Allelic Dosage-corrected Allele-Specific human TF binding sites) database [32].

TF	Effect Size G Allele	Effect Size A Allele	FDR G Allele	FDR A Allele	Mean BAD	Motif Fold Change	Motif Concordance
GABPB1	1.54	n/a	3.7 × 10^−8^	1.00	3.00	n/a	n/a
MNT	2.64	n/a	2.2 × 10^−5^	1.00	2.33	n/a	no hit
NR2C1	2.27	n/a	4.0 × 10^−5^	1.00	2.75	n/a	no hit
ZBTB40	2.64	n/a	6.7 × 10^−5^	1.00	3.00	n/a	no hit
E2F1	3.61	n/a	1.6 × 10^−4^	1.00	2.00	n/a	no hit
NR2F1	2.31	n/a	2.2 × 10^−3^	1.00	2.50	n/a	no hit
ATF3	2.99	−5.21	3.9 × 10^−3^	1.00	2.00	n/a	no hit
RFX1	2.23	−0.86	4.3 × 10^−3^	1.00	2.00	n/a	no hit
VEZF1	2.98	n/a	7.9 × 10^−3^	1.00	3.00	−3.30	concordant
FOXK2	2.59	−0.14	7.9 × 10^−3^	1.00	2.00	n/a	no hit
CREM	2.82	−5.34	0.03	1.00	2.00	n/a	no hit
ATF1	2.30	n/a	0.03	1.00	2.50	n/a	no hit
ZBTB2	2.86	−5.08	0.04	1.00	2.00	n/a	no hit

Legend: BAD, Background Allelic Dosage; FDR, False Discovery Rate; TF, transcription factor; no hit, no match to the canonical TF motif.

**Table 7:** *CYBA* gene expression in the aorta, tibial artery and left ventricle, depending on the genotypes of the rs13306296 and rs4673 SNPs, based on the data from the GTEx Portal [33].

SNP	Genotype	n	Tissue	Median	NES	T Statistic	*p*	*p* Threshold
rs13306296	GG	195	Aorta	0.029	−0.18	−8.4	5.4 × 10^−16^	8.3 × 10^−5^
(–536 C>T)	GA	218		0.037				
	AA	59		−0.238				
	GG	300	Tibial	0.140	−0.10	−7.3	1.2 × 10^−12^	1.2 × 10^−4^
	GA	297	artery	−0.102				
	AA	92		−0.135				
	GG	184	Left	0.236	−0.18	−8.0	2.1 × 10^−14^	6.2 × 10^−5^
	GA	208	ventricle	−0.073				
	AA	58		−0.089				
	GG	192	Atrial	0.298	−0.17	−5.6	3.8 × 10^−8^	7.8 × 10^−5^
	GA	203	appendage	−0.134				
	AA	95		−0.267				
rs4673	AA	72	Aorta	−0.157	−0.13	−6.0	4.3 × 10^−9^	8.3 × 10^−5^
(214 C>T,	GA	209		0.136				
C242T)	GG	191		−0.146				
	AA	105	Tibial	0.113	−0.06	−4.0	6.9 × 10^−5^	1.2 × 10^−4^
	GA	302	artery	0.133				
	GG	282		−0.177				

Legend: NES, normalized effect size; SNP, single nucleotide polymorphism.

**Table 8:** Transcription factors (TFs) preferentially binding to the G allele of rs13306296.

TF	Full Name	Main Function
GABPB1	GA binding protein transcription factor subunit beta 1	Subunit α of the GA-binding protein (GABP) transcriptionally active complex, an obligate heterotetramer. It regulates genes that are intimately involved in cell cycle control, protein synthesis, and cellular metabolism, mitochondrial metabolism, mitochondrial biogenesis and beige adipogenesis [37,38].
MNT	MAX network transcriptional repressor	Forms heterodimers with MAX or MLX proteins. Negatively regulates transcription by RNA polymerase II, cell cycle, apoptotic signaling, cellular senescence and the NF-κB pathway. Competes with MYC for the same DNA sites to block genes [39,40]. The *CYBA* gene has E-box sequences, and it can be directly bound and modulated by the MYC/MAX/MNT network, especially under conditions of cellular stress.
NR2C1	Nuclear receptor subfamily 2 group C member 1	Nuclear transcription factor belonging to the steroid and thyroid hormone receptor superfamily. Its primary functions include regulating hemoglobin synthesis, maintaining stem cell pluripotency, and participating in tissue differentiation, including preadipocyte proliferation. It influences the expression of the main adipose tissue regulator, PPARγ [41].
ZBTB40	Zinc finger and BTB domain containing protein 40	It determines how intensively stem cells differentiate into adipose tissue (via PPARγ) and protects telomeres against the damaging effects of oxidative stress [42].
E2F1	E2F transcription factor 1	A major regulator of the cell cycle, the transition from G1 to S phase, DNA replication, and apoptosis. It regulates adipocyte differentiation and adipogenesis, as well as oxidative metabolism [43,44].
NR2F1	Nuclear receptor subfamily 2 group F member 1	It plays a key role in neurogenesis and brain development, neuronal plasticity, and oncogenesis. It modulates lipid transport efficiency (by altering MTP protein expression and controlling TC and TG levels) and local tissue response to oxidative stress [45,46].
ATF3	Activating transcription factor 3	Belongs to the ATF/cAMP response element-binding family. Promotes HDL uptake in hepatocytes, facilitates reverse cholesterol transport from macrophages, and reduces intestinal lipid absorption [47], while in adipocytes it suppresses adipogenesis [48].
RFX1	Regulatory factor X1	Context-dependent TF whose effects on gene expression are determined by the specific target gene and are not necessarily dependent on the cellular environment [49]. Inhibits the transcription of the *CD36* gene, a receptor involved in the uptake of oxLDL by macrophages. A decrease in RFX1 levels leads to increased CD36 expression, greater uptake of oxLDL, and lipid accumulation in macrophages, promoting the formation of foam cells and the development of atherosclerotic lesions [50].
VEZF1	Vascular endothelial zinc finger 1	A zinc finger transcription factor involved in endothelial cell differentiation, vascular development, and angiogenesis [51,52]. Its silencing increases oxidative stress and promotes lipid peroxidation-associated ferroptosis [53].
FOXK2	Forkhead box K2	Contains a conserved forkhead DNA-binding domain and binds specific DNA motifs to regulate a wide range of biological processes including metabolism, cell cycle progression, proliferation, survival, differentiation and apoptosis [54]. Overexpression of *FOXK2* promotes adipogenic differentiation [55].
CREM	cAMP responsive element modulator	A member of the cAMP response element-binding protein (CREB) TF family. It is best known for its involvement in spermatogenesis; however, it also participates in the early stages of adipocyte differentiation [56,57].
ATF1	Activating transcription factor 1	Belongs to the ATF subfamily and bZIP (basic-region leucine zipper) family. It modulates gene expression in response to extracellular signals [58]. ATF1 contributes to adipocyte differentiation together with CREB [59].
ZBTB2	Zinc finger and BTB domain-containing protein 2	Plays a crucial role in many cellular processes such as cellular development, proliferation, differentiation, cancer and pluripotency [60]. ZBTB2 controls the genes of the p53 pathway and reprograms metabolic pathways favoring glycolysis over the Krebs cycle, thereby suppressing oxidative metabolism [61].

**Table 9:** Formulas for lipid-derived atherogenic indices used in the present study.

**Index**	**Units**	**Formula**
CRI-I	[mg/dL]	TC/HDL-C
CRI-II	[mg/dL]	LDL-C/HDL-C
LCI	[mmol/L]	(TC × TG × LDL-C)/HDL-C
AC	[mmol/L]	(TC–HDL-C)/HDL-C
AIP	[mg/dL]	log _10_ (TG/HDL-C)
TG/HDL-C	[mg/dL]	TG/HDL-C

## Data Availability

The data supporting the findings of this study are available within the article. Individual-level data are not publicly available due to ethical and privacy restrictions but may be obtained from the corresponding author upon reasonable request and subject to approval by the relevant ethics committee.

## Supplementary Materials

The following supporting information can be downloaded at https://www.mdpi.com/article/10.3390/ijms27208975/s1.

## Abbreviations

The following abbreviations are used in this manuscript:

ABCA1	ATP-binding cassette subfamily A member 1
ABCG	ATP-binding cassette subfamily G
AC	Atherogenic coefficient
ADASTRA	Allelic dosage-corrected allele-specific human TF binding sites database
AIP	Atherogenic index of plasma
Apo A-I	Apolipoprotein A-I
ASB	Allele-specific binding
ATF	Activating transcription factor
BMI	Body mass index
CAD	Coronary artery disease
CE	Cholesteryl ester
CETP	Cholesteryl ester transfer protein
CRI	Castelli’s risk index
CREM	cAMP responsive element modulator
*CYBA*	Cytochrome b(-245), alpha subunit gene
DUOX	Dual oxidase
E2F1	E2F transcription factor 1
FOXK2	Forkhead box K2
GABPB1	GA binding protein transcription factor subunit beta 1
GLGC	Global Lipids Genetics Consortium
GRCh38	Genome reference consortium Human Build 38 organism: Homo sapiens
HDL	High-density lipoprotein
HDL-C	High-density lipoprotein cholesterol
HMG-CoA	3-hydroxy-3-methylglutaryl-CoA
HWE	Hardy–Weinberg equilibrium
LCAT	Lecithin–cholesterol acyltransferase
LCI	Lipid combination index
LDL	Low-density lipoprotein
LDL-C	Low-density lipoprotein cholesterol
LDLR	Low-density lipoprotein receptor
MAF	Minor allele frequency
MI	Myocardial infarction
MNT	MAX network transcriptional repressor
NADPH	Nicotinamide adenine dinucleotide phosphate, reduced
NOX	NADPH oxidase
NR2C1	Nuclear receptor subfamily 2 group C member 1
NR2F1	Nuclear receptor subfamily 2 group F member 1
oxHDL	Oxidized HDL
oxLDL	Oxidized LDL
PCR	Polymerase chain reaction
Phox	Phagocyte oxidase
RCT	Reverse cholesterol transport
RFX1	Regulatory factor X1
ROS	Reactive oxygen species
SNP	Single nucleotide polymorphism
SR-B1	Scavenger receptor class B type 1
SREBP	Sterol regulatory element-binding protein
STROBE	Strengthening the reporting of observational studies in epidemiology
TC	Total cholesterol
TF	Transcription factor
TG	Triacylglycerols
VEZF1	Vascular endothelial zinc finger 1
ZBTB2	Zinc finger and BTB domain-containing protein 2
ZBTB40	Zinc finger and BTB domain-containing protein 40
