# Supplementary material for: CYBA Gene Polymorphisms, Lipid Parameters and Atherogenic Indices: A Cohort Study of Healthy Individuals Without Prior Lipid-Lowering Therapy
Source: Int J Mol Sci. 2026 Oct 10;27(20):8975. doi: 10.3390/ijms27208975 (PMC13704446; doi:10.3390/ijms27208975)
Supplement: Supplementary file 1 [file ijms-27-08975-s001.zip › Table S1.pdf]

Table S1. Lipid parameters according to genotypes of the *CYBA* gene polymorphisms (additive model).

Parameter	rs7195830 (*49 A>G)						*P* value			
	AA, n=51		AG, n=100		GG, n=65					
	Median	±QD	Median	±QD	Median	±QD	Kruskal-Wallis test	AA vs AG	AA vs GG	AG vs GG
TC [mg/dl]	206.00	35.00	198.00	30.00	206.00	25.50	0.139			
HDL-C [mg/dl]	51.00	14.00	43.00	8.00	46.00	9.00	**0.001***	**0.000***	**0.012**	1.000
LDL-C [mg/dl]	123.80	37.50	122.00	31.30	132.20	24.70	0.143			
TG [mg/dl]	113.00	41.50	132.00	45.50	124.00	42.00	**0.048**	**0.047**	0.760	0.614
CRI-I [mg/dl]	4.06	1.02	4.57	1.09	4.64	0.94	**0.032**	0.053	0.058	1.000
CRI-II [mg/dl]	2.45	0.88	2.82	0.97	2.93	0.81	0.072			
LCI [mmol/l]	16.90	11.98	27.04	17.45	24.64	12.98	0.078			
AC [mmol/l]	3.06	1.02	3.57	1.09	3.64	0.94	**0.032**	0.053	0.058	1.000
AIP [mg/dl]	0.34	0.25	0.52	0.17	0.46	0.19	**0.001***	**0.001***	0.090	0.484
TG/HDL-C [mg/dl]	2.19	1.16	3.33	1.21	2.90	1.13	**0.001***	**0.001***	0.090	0.484
**Parameter**	**rs1049255 (*24 A>G, A640G)**						***P* value**			
	**TT, n=64**		**TC, n=106**		**CC, n=49**					
	**Median**	**±QD**	**Median**	**±QD**	**Median**	**±QD**	**Kruskal-Wallis test**	**TT vs TC**	**TT vs CC**	**TC vs CC**
TC [mg/dl]	202.50	24.75	204.00	30.50	197.00	29.50	0.993			
HDL-C [mg/dl]	43.00	7.00	45.00	9.75	51.00	14.50	**0.006***	1.000	**0.007***	**0.022**
LDL-C [mg/dl]	128.30	25.80	128.00	27.70	113.50	32.90	0.327			
TG [mg/dl]	124.00	42.75	125.00	43.00	124.00	37.00	0.787			
CRI-I [mg/dl]	4.59	0.91	4.48	1.11	4.02	1.07	0.055			
CRI-II [mg/dl]	2.93	0.81	2.82	0.96	2.34	0.90	0.051			
LCI [mmol/l]	23.56	13.52	24.37	17.06	18.63	13.25	0.366			
AC [mmol/l]	3.59	0.91	3.48	1.11	3.02	1.07	0.054			
AIP [mg/dl]	0.41	0.19	0.50	0.17	0.38	0.21	0.202			
TG/HDL-C [mg/dl]	2.58	1.22	3.19	1.15	2.39	1.12	0.202			

Table S1. Lipid parameters according to genotypes of the *CYBA* gene polymorphisms (additive model), continued.

**Parameter**	**rs4673 (214 C>T C242T)**						**P value**			
	**GG, n=94**		**GA, n=91**		**AA, n=28**					
	**Median**	**±QD**	**Median**	**±QD**	**Median**	**±QD**	**Kruskal-Wallis test**	**GG vs GA**	**GG vs AA**	**GA vs AA**
TC [mg/dl]	205.50	26.00	196.00	31.50	200.50	20.25	0.823			
HDL-C [mg/dl]	47.50	9.00	44.00	9.50	42.00	8.25	0.093			
LDL-C [mg/dl]	124.90	30.90	122.00	30.00	128.00	21.60	0.691			
TG [mg/dl]	119.00	41.00	125.50	44.50	145.00	37.75	0.844			
CRI-I [mg/dl]	4.33	0.87	4.51	1.12	5.16	1.02	0.126			
CRI-II [mg/dl]	2.63	0.80	2.80	0.99	3.41	0.78	0.104			
LCI [mmol/l]	19.34	14.53	25.06	17.78	29.40	12.72	0.660			
AC [mmol/l]	3.33	0.87	3.51	1.12	4.16	1.02	0.126			
AIP [mg/dl]	0.38	0.21	0.48	0.21	0.51	0.14	0.391			
TG/HDL-C [mg/dl]	2.40	1.26	3.03	1.33	3.23	1.00	0.391			
**Parameter**	**rs13306296 (–536 C>T)**						**P value**			
	**GG, n=93**		**GA, n=94**		**AA, n=25**					
	**Median**	**±QD**	**Median**	**±QD**	**Median**	**±QD**	**Kruskal-Wallis test**	**GG vs GA**	**GG vs AA**	**GA vs AA**
TC [mg/dl]	206.00	27.50	197.00	25.50	199.00	32.50	0.181			
HDL-C [mg/dl]	46.00	6.50	49.00	12.00	49.00	14.50	0.151			
LDL-C [mg/dl]	129.20	26.40	124.00	26.00	104.20	27.30	**0.014**	0.474	**0.012**	0.148
TG [mg/dl]	145.00	43.00	113.00	33.00	99.00	27.50	0.088			
CRI-I [mg/dl]	4.74	0.93	4.29	1.23	3.57	1.13	**0.008***	0.634	**0.006***	0.070
CRI-II [mg/dl]	3.02	0.87	2.70	0.97	1.97	0.94	**0.003***	0.888	**0.002***	**0.020**
LCI [mmol/l]	30.22	17.89	20.66	13.51	13.99	7.06	**0.016**	0.255	**0.018**	0.351
AC [mmol/l]	3.74	0.93	3.29	1.23	2.57	1.13	**0.008***	0.633	**0.006***	0.069
AIP [mg/dl]	0.49	0.16	0.43	0.21	0.32	0.16	0.108			
TG/HDL-C [mg/dl]	3.08	1.12	2.67	1.23	2.10	0.90	0.108			

Table S1. Lipid parameters according to genotypes of the *CYBA* gene polymorphisms (additive model), continued.

**Parameter**	**rs9932581 (–930 A>G)**						***P* value**			
	**TT, n=41**		**TC, n=104**		**CC, n=73**					
	**Median**	**±QD**	**Median**	**±QD**	**Median**	**±QD**	**Kruskal-Wallis test**	**TT vs TC**	**TT vs CC**	**TC vs CC**
TC [mg/dl]	208.00	28.00	200.00	28.50	202.00	29.50	0.982			
HDL-C [mg/dl]	45.00	10.50	48.50	12.00	44.00	7.00	0.111			
LDL-C [mg/dl]	125.60	25.70	119.00	31.25	132.40	25.10	0.332			
TG [mg/dl]	126.00	36.00	124.00	39.50	120.00	47.00	0.496			
CRI-I [mg/dl]	4.51	0.92	4.42	1.08	4.43	1.11	0.139			
CRI-II [mg/dl]	2.82	0.85	2.66	0.97	2.82	0.98	0.135			
LCI [mmol/l]	26.25	15.60	19.04	14.36	26.82	16.17	0.175			
AC [mmol/l]	3.51	0.92	3.42	1.08	3.43	1.11	0.139			
AIP [mg/dl]	0.52	0.17	0.38	0.24	0.52	0.15	0.070			
TG/HDL-C [mg/dl]	3.31	1.01	2.42	1.40	3.33	1.10	0.070			

Legend: AC, atherogenic coefficient; AIP, atherogenic index of plasma; CRI-I, Castelli's risk index I; CRI-II, Castelli's risk index II; HDL-C, high density lipoprotein; LCI, lipid combination index; LDL-C, low density lipoprotein; TC, total cholesterol; TG, triglycerides; TG/HDL-C, triglyceride-to-HDL-C ratio; QD, quartile deviation. *, differences remaining significant after Bonferroni-Hochberg correction for multiple comparisons (threshold of significance for additive model: $p \leq 0.008$ in Kruskal-Wallis test).
