# Supplementary material for: CYBA Gene Polymorphisms, Lipid Parameters and Atherogenic Indices: A Cohort Study of Healthy Individuals Without Prior Lipid-Lowering Therapy
Source: Int J Mol Sci. 2026 Oct 10;27(20):8975. doi: 10.3390/ijms27208975 (PMC13704446; doi:10.3390/ijms27208975)
Supplement: Supplementary file 1 [file ijms-27-08975-s001.zip › Table S2.pdf]

Table S2. Lipid parameters according to genotypes of the *CYBA* gene polymorphisms (dominant/recessive model).

Parameter	rs7195830 (*49 A>G)				*P*
	AA, n=51		AG/GG, n=164		
	Median	±QD	Median	±QD	
TC [mg/dl]	206.00	35.00	200.50	27.00	0.254
HDL-C [mg/dl]	51.00	14.00	44.00	8.50	**0.000***
LDL-C [mg/dl]	123.80	37.50	126.00	25.30	0.847
TG [mg/dl]	113.00	41.50	126.00	42.00	**0.037**
CRI-I [mg/dl]	4.06	1.02	4.58	1.04	**0.009**
CRI-II [mg/dl]	2.45	0.88	2.89	0.92	**0.029**
LCI [mmol/l]	16.90	11.98	26.76	15.68	**0.025**
AC [mmol/l]	3.06	1.02	3.58	1.04	**0.009**
AIP [mg/dl]	0.34	0.25	0.50	0.18	**0.001***
TG/HDL-C [mg/dl]	2.19	1.16	3.14	1.22	**0.001***
**Parameter**	**rs1049255 (*24 A>G, A640G)**				***P***
	**TT/TC, n=170**		**CC, n=50**		
	**Median**	**±QD**	**Median**	**±QD**	
TC [mg/dl]	203.00	28.50	197.00	29.50	0.972
HDL-C [mg/dl]	44.50	8.50	51.00	14.50	**0.002***
LDL-C [mg/dl]	128.00	26.10	113.50	32.90	0.172
TG [mg/dl]	124.00	43.50	124.00	37.00	0.646
CRI-I [mg/dl]	4.50	1.03	4.02	1.07	**0.019**
CRI-II [mg/dl]	2.85	0.90	2.34	0.90	**0.020**
LCI [mmol/l]	23.94	15.90	18.63	13.25	0.157
AC [mmol/l]	3.50	1.03	3.02	1.07	**0.019**
AIP [mg/dl]	0.48	0.18	0.38	0.21	0.078
TG/HDL-C [mg/dl]	3.05	1.16	2.39	1.12	0.078
**Parameter**	**rs4673 (214 C>T, C242T)**				***P***
	**GG, n=94**		**GA/AA, n=119**		
	**Median**	**±QD**	**Median**	**±QD**	
TC [mg/dl]	205.50	26.00	200.00	29.00	0.556
HDL-C [mg/dl]	47.50	9.00	44.00	10.00	**0.027**
LDL-C [mg/dl]	124.90	30.90	126.00	26.70	0.648
TG [mg/dl]	119.00	41.00	130.00	44.50	0.561
CRI-I [mg/dl]	4.33	0.87	4.74	1.11	0.083
CRI-II [mg/dl]	2.63	0.80	2.93	0.96	0.073
LCI [mmol/l]	19.34	14.53	25.73	16.20	0.399
AC [mmol/l]	3.33	0.87	3.74	1.11	0.083
AIP [mg/dl]	0.38	0.21	0.48	0.18	0.180
TG/HDL-C [mg/dl]	2.40	1.26	3.05	1.20	0.180

Legend: AC, atherogenic coefficient; AIP, atherogenic index of plasma; CRI-I, Castelli's risk index I; CRI-II, Castelli's risk index II; HDL-C, high density lipoprotein; LCI, lipid combination index; LDL-C, low density lipoprotein; TC, total cholesterol; TG, triglycerides; TG/HDL-C, triglyceride-to-HDL-C ratio; QD, quartile deviation. *, differences remaining significant after Bonferroni-Hochberg correction for multiple comparisons (threshold of significance for recessive/dominant model: $P \leq 0.004$).

Table S2. Lipid parameters according to genotypes of the *CYBA* gene polymorphisms (dominant/recessive model), continued.

Parameter	rs13306296 (–536 C>T)				*P*
	GG/GA, n=187		AA, n=25		
	Median	±QD	Median	±QD	
TC [mg/dl]	202.00	27.50	199.00	32.50	0.195
HDL-C [mg/dl]	46.00	9.50	49.00	14.50	0.080
LDL-C [mg/dl]	128.00	27.10	104.20	27.30	**0.010**
TG [mg/dl]	126.00	44.00	99.00	27.50	0.270
CRI-I [mg/dl]	4.49	1.04	3.57	1.13	**0.004***
CRI-II [mg/dl]	2.82	0.91	1.97	0.94	**0.001***
LCI [mmol/l]	24.64	15.07	13.99	7.06	**0.021**
AC [mmol/l]	3.49	1.04	2.57	1.13	**0.004***
AIP [mg/dl]	0.46	0.21	0.32	0.16	0.107
TG/HDL-C [mg/dl]	2.90	1.29	2.10	0.90	0.107
**Parameter**	**rs72811418 (–675 C>T)**				***P***
	**AA/AT, n=28**		**TT, n=189**		
	**Median**	**±QD**	**Median**	**±QD**	
TC [mg/dl]	208.00	33.50	200.00	27.00	0.456
HDL-C [mg/dl]	41.00	9.50	47.00	9.50	**0.020**
LDL-C [mg/dl]	131.20	31.25	125.40	28.75	0.271
TG [mg/dl]	140.00	40.50	123.00	44.50	0.460
CRI-I [mg/dl]	5.20	1.18	4.39	1.08	**0.022**
CRI-II [mg/dl]	3.48	0.95	2.70	0.96	**0.018**
LCI [mmol/l]	32.09	26.65	21.78	14.80	0.085
AC [mmol/l]	4.20	1.18	3.39	1.08	**0.022**
AIP [mg/dl]	0.53	0.14	0.41	0.21	0.074
TG/HDL-C [mg/dl]	3.39	1.08	2.60	1.28	0.074
**Parameter**	**rs9932581 (–930 A>G)**				***P***
	**TT/TC, n=145**		**CC, n=74**		
	**Median**	**±QD**	**Median**	**±QD**	
TC [mg/dl]	201.00	27.75	202.00	29.50	0.900
HDL-C [mg/dl]	47.00	11.00	44.00	7.00	0.064
LDL-C [mg/dl]	119.60	27.60	132.40	25.10	0.168
TG [mg/dl]	124.50	37.75	120.00	47.00	0.238
CRI-I [mg/dl]	4.49	1.12	4.43	1.11	0.067
CRI-II [mg/dl]	2.71	0.94	2.82	0.98	0.074
LCI [mmol/l]	21.01	14.97	26.82	16.17	0.077
AC [mmol/l]	3.49	1.12	3.43	1.11	0.067
AIP [mg/dl]	0.41	0.21	0.52	0.15	**0.032**
TG/HDL-C [mg/dl]	2.58	1.22	3.33	1.10	**0.032**

Legend: AC, atherogenic coefficient; AIP, atherogenic index of plasma; CRI-I, Castelli's risk index I; CRI-II, Castelli's risk index II; HDL-C, high density lipoprotein; LCI, lipid combination index; LDL-C, low density lipoprotein; TC, total cholesterol; TG, triglycerides; TG/HDL-C, triglyceride-to-HDL-C ratio; QD, quartile deviation. *, differences remaining significant after Bonferroni-Hochberg correction for multiple comparisons (threshold of significance for recessive/dominant model: $P \leq 0.004$).
