# Supplementary material for: CYBA Gene Polymorphisms, Lipid Parameters and Atherogenic Indices: A Cohort Study of Healthy Individuals Without Prior Lipid-Lowering Therapy
Source: Int J Mol Sci. 2026 Oct 10;27(20):8975. doi: 10.3390/ijms27208975 (PMC13704446; doi:10.3390/ijms27208975)
Supplement: Supplementary file 1 [file ijms-27-08975-s001.zip › Table S3.pdf]

Table S3. Predictors of lipid parameters in additive model (multivariable regression analysis);

Lipid parameter	Predictor	variant	B	SE B	Lower 95% CI B	Upper 95% CI B	β	SE β	Lower 95% CI β	Upper 95% CI β	t	P
TC	rs13306296	GG	16.11	5.93	4.40	27.81	0.34	0.13	0.09	0.59	2.72	0.007
	rs13306296	AA	–18.32	7.59	–33.31	–3.33	–0.27	0.11	–0.49	–0.05	–2.41	0.017
	Age		1.31	0.51	0.30	2.31	0.19	0.07	0.04	0.33	2.56	0.011
	BMI		1.98	0.84	0.33	3.63	0.18	0.07	0.03	0.33	2.36	0.019
HDL	rs7195830	AA	7.40	2.93	1.63	13.18	0.40	0.16	0.09	0.71	2.53	0.012
	rs1049255	TT	–5.99	2.92	–11.77	–0.22	–0.32	0.16	–0.63	–0.01	–2.05	0.042
LDL	rs13306296	GG	17.47	5.53	6.54	28.39	0.40	0.13	0.15	0.65	3.16	0.002
	Age		1.26	0.48	0.32	2.20	0.20	0.07	0.05	0.34	2.65	0.009
	rs4673	AA	11.06	5.46	0.28	21.85	0.18	0.09	0.00	0.36	2.03	0.044
	rs13306296	AA	–22.97	7.08	–36.94	–9.00	–0.36	0.11	–0.59	–0.14	–3.25	0.001
TG	BMI		5.95	1.57	2.86	9.05	0.28	0.07	0.14	0.43	3.80	0.000
CRI-I	Age		0.06	0.02	0.02	0.10	0.22	0.07	0.08	0.37	3.07	0.003
	rs13306296	AA	–0.75	0.30	–1.33	–0.16	–0.28	0.11	–0.50	–0.06	–2.53	0.012
CRI-II	Age		0.05	0.02	0.01	0.08	0.21	0.07	0.06	0.35	2.82	0.005
	rs13306296	AA	–0.69	0.25	–1.19	–0.19	–0.31	0.11	–0.53	–0.09	–2.74	0.007
LCI	rs13306296	GG	10.74	4.58	1.69	19.79	0.29	0.12	0.05	0.54	2.34	0.020
	Age		1.23	0.40	0.44	2.02	0.22	0.07	0.08	0.37	3.07	0.002
	BMI		1.70	0.64	0.43	2.97	0.20	0.07	0.05	0.34	2.63	0.009
	rs13306296	AA	–11.98	5.85	–23.53	–0.43	–0.23	0.11	–0.45	–0.01	–2.05	0.042
AC	Age		0.06	0.02	0.02	0.10	0.22	0.07	0.08	0.37	3.07	0.003
	rs13306296 CYBA	AA	–0.75	0.30	–1.33	–0.16	–0.28	0.11	–0.50	–0.06	–2.53	0.012
AIP	BMI		0.02	0.01	0.01	0.03	0.23	0.07	0.08	0.38	3.13	0.002
	Age		0.01	0.00	0.00	0.02	0.18	0.07	0.04	0.33	2.56	0.011
TG/HDL	BMI		0.12	0.04	0.04	0.21	0.21	0.08	0.06	0.36	2.79	0.006
	Age		0.07	0.03	0.01	0.12	0.18	0.07	0.04	0.33	2.45	0.015

Legend: B, adjusted unstandardized regression coefficient (effect in lipid parameter units); β, standardized regression coefficient (to compare the strength of individual predictors); CI, confidence interval. AC, atherogenic coefficient; AIP, atherogenic index of plasma; BMI, body mass index; CRI-I, Castelli's risk index I; CRI-II, Castelli's risk index II; HDL-C, high-density lipoprotein cholesterol; LCI, lipid combination index; LDL-C, low-density lipoprotein cholesterol; TC, total cholesterol; TG, triacylglycerols; TG/HDL-C, triglyceride-to-HDL-C ratio.
