# Supplementary material for: CYBA Gene Polymorphisms, Lipid Parameters and Atherogenic Indices: A Cohort Study of Healthy Individuals Without Prior Lipid-Lowering Therapy
Source: Int J Mol Sci. 2026 Oct 10;27(20):8975. doi: 10.3390/ijms27208975 (PMC13704446; doi:10.3390/ijms27208975)
Supplement: Supplementary file 1 [file ijms-27-08975-s001.zip › Table S4.pdf]

Table S4. Predictors of lipid parameters in dominant/recessive model (multivariable regression analysis);

Lipid parameter	Predictor	variant	B	SE B	Lower 95% CI B	Upper 95% CI B	β	SE β	Lower 95% CI β	Upper 95% CI β	t	P
TC	BMI		2.13	0.82	0.50	3.76	0.19	0.07	0.05	0.34	2.59	0.011
	rs13306296	AA	–13.79	5.59	–24.83	–2.76	–0.19	0.08	–0.34	–0.04	–2.47	0.015
	Age		1.26	0.51	0.26	2.27	0.18	0.07	0.04	0.33	2.48	0.014
HDL-C	Lack of predictors											
LDL-C	rs13306296	AA	–17.12	5.18	–27.35	–6.89	–0.25	0.08	–0.41	–0.10	–3.30	0.001
	Age		1.21	0.47	0.27	2.14	0.19	0.07	0.04	0.33	2.55	0.012
TG	BMI		6.45	1.54	3.42	9.48	0.31	0.07	0.16	0.45	4.20	0.000
	rs4673	AA/GA	13.93	7.00	0.12	27.74	0.16	0.08	0.00	0.33	1.99	0.048
CRI-I	Age		0.06	0.02	0.02	0.10	0.22	0.07	0.08	0.36	3.01	0.003
	rs72811418	TT	–0.62	0.22	–1.05	–0.19	–0.22	0.08	–0.38	–0.07	–2.82	0.005
	rs13306296	AA	–0.52	0.22	–0.95	–0.09	–0.18	0.08	–0.34	–0.03	–2.39	0.018
CRI-II	rs72811418	TT	–0.56	0.19	–0.92	–0.19	–0.24	0.08	–0.39	–0.08	–3.01	0.003
	rs13306296	AA	–0.50	0.18	–0.86	–0.13	–0.21	0.08	–0.36	–0.06	–2.70	0.008
	Age		0.05	0.02	0.01	0.08	0.20	0.07	0.06	0.35	2.80	0.006
LCI	Age		1.18	0.40	0.40	1.97	0.22	0.07	0.07	0.36	2.98	0.003
	BMI		1.92	0.63	0.67	3.16	0.22	0.07	0.08	0.37	3.03	0.003
	rs9932581	CC	6.11	2.67	0.84	11.37	0.17	0.07	0.02	0.32	2.29	0.023
AC	Age		0.06	0.02	0.02	0.10	0.22	0.07	0.08	0.36	3.01	0.003
	rs72811418	TT	–0.62	0.22	–1.05	–0.19	–0.22	0.08	–0.38	–0.07	–2.82	0.005
	rs13306296	AA	–0.52	0.22	–0.95	–0.09	–0.18	0.08	–0.34	–0.03	–2.39	0.018
AIP	Age		0.01	0.00	0.00	0.02	0.17	0.07	0.03	0.32	2.38	0.018
	BMI		0.02	0.01	0.01	0.03	0.25	0.07	0.11	0.40	3.45	0.001
TG/HDL	Age		0.06	0.03	0.01	0.12	0.17	0.07	0.03	0.32	2.34	0.021

Legend: B, adjusted unstandardized regression coefficient (effect in lipid parameter units); β, standardized regression coefficient (to compare the strength of individual predictors); CI, confidence interval. AC, atherogenic coefficient; AIP, atherogenic index of plasma; BMI, body mass index; CRI-I, Castelli's risk index I; CRI-II, Castelli's risk index II; HDL-C, high-density lipoprotein cholesterol; LCI, lipid combination index; LDL-C, low-density lipoprotein cholesterol; TC, total cholesterol; TG, triacylglycerols; TG/HDL-C, triglyceride-to-HDL-C ratio.
