# Supplementary material for: CYBA Gene Polymorphisms, Lipid Parameters and Atherogenic Indices: A Cohort Study of Healthy Individuals Without Prior Lipid-Lowering Therapy
Source: Int J Mol Sci. 2026 Oct 10;27(20):8975. doi: 10.3390/ijms27208975 (PMC13704446; doi:10.3390/ijms27208975)
Supplement: Supplementary file 1 [file ijms-27-08975-s001.zip › Table S5.pdf]

Table S5. GLGC (Global Lipids Genetics Consortium) summary statistics for *CYBA* variants in the context of lipid parameters [89].

SNP	Trait	REF	ALT (effect)	ALT AF	N	β (ALT)	SE	P	Higher-value allele	Present study *
rs7195830	HDL-C	G	A	0.371	1,487,420	+0.0013	0.0013	0.310	A	Lower HDL-C, higher AIP and TG/HDL-C in GG/AG vs AA
	LDL-C	G	A	0.371	1,452,940	-0.0025	0.0013	0.061	G	
	TC	G	A	0.372	1,567,720	-0.0038	0.0013	3.02×10^{-3}	G	
	logTG	G	A	0.371	1,480,440	-0.0004	0.0013	1.35×10^{-4}	G	
rs1049255	HDL-C	C	T	0.494	1,444,690	-0.0005	0.0013	0.711	C	Lower HDL-C in TT
	LDL-C	C	T	0.494	1,432,270	+0.0020	0.0013	0.124	T	
	TC	C	T	0.494	1,524,900	+0.0032	0.0013	0.011	T	
	logTG	C	T	0.494	1,464,120	+0.0026	0.0013	0.040	T	
rs4673	HDL-C	G	A	0.322	1,467,940	-0.0011	0.0014	0.399	G	Higher LDL-C in AA, higher TG in AA/GA
	LDL-C	G	A	0.322	1,435,390	+0.0050	0.0014	3.30×10^{-4}	A	
	TC	G	A	0.322	1,548,120	+0.0051	0.0013	1.09×10^{-4}	A	
	logTG	G	A	0.322	1,461,990	+0.0049	0.0014	3.37×10^{-4}	A	
rs13306296	HDL-C	G	A	0.339	1,458,900	+0.0012	0.0014	0.378	A	Higher TC, LDL-C, CRI-I, CRI-II, LCI, AC in GG/GA vs AA. Additive effect: descending GG>GA>AA
	LDL-C	G	A	0.340	1,429,640	-0.0035	0.0014	0.012	G	
	TC	G	A	0.341	1,539,060	-0.0033	0.0014	0.014	G	
	logTG	G	A	0.340	1,457,420	-0.0044	0.0014	0.002	G	
rs72811418	HDL-C	A	T	0.0847	1,418,920	-0.0011	0.0024	0.662	A	Lower CRI-I, CRI-II, AC in TT
	LDL-C	A	T	0.085	1,391,660	-0.0013	0.0024	0.598	A	
	TC	A	T	0.0847	1,498,190	-0.0002	0.0023	0.936	A	
	logTG	A	T	0.0848	1,417,610	+0.0012	0.0024	0.624	T	
rs16966671	HDL-C	C	G	0.460	1,306,490	-0.0023	0.0015	0.121	C	no effect in the present study
	LDL-C	C	G	0.460	1,314,720	+0.0026	0.0015	0.073	G	
	TC	C	G	0.459	1,383,260	+0.0033	0.0014	0.021	G	
	logTG	C	G	0.461	1,347,950	+0.0044	0.0014	0.002	G	
rs9932581	HDL-C	C	T	0.393	1,337,420	+0.0002	0.0015	0.902	T	Higher LCI in CC
	LDL-C	C	T	0.393	1,346,800	-0.0029	0.0015	0.055	C	
	TC	C	T	0.393	1,402,270	-0.0027	0.0015	0.061	C	
	logTG	C	T	0.393	1,374,300	-0.0009	0.0015	0.538	C	

Legend: ALT, the effect allele in the GLGC files; β, the METAL effect estimate for ALT. Higher-value allele was assigned from the sign of β: ALT when $\beta > 0$ and REF when $\beta < 0$. ALT AF denotes pooled ALT allele frequency. TG was analyzed as logTG in the GLGC dataset. Bold P values indicate nominal $P < 0.05$; * differences significant after correction for multiple comparisons.
